# Menopausal Hormone Therapy Use Among Postmenopausal Women

**DOI:** 10.1001/jamahealthforum.2024.3128

**Published:** 2024-09-27

**Authors:** Lin Yang, Adetunji T. Toriola

**Affiliations:** 1 Department of Cancer Epidemiology and Prevention Research, Cancer Research and Analytics, Cancer Care Alberta, Alberta Health Services, Calgary, Alberta, Canada; 2 Department of Oncology, Cumming School of Medicine, University of Calgary, Calgary, Alberta, Canada; 3 Department of Community Health Sciences, Cumming School of Medicine, University of Calgary, Calgary, Alberta, Canada; 4 Division of Public Health Sciences, Department of Surgery, Washington University School of Medicine, St Louis, Missouri; 5 Alvin J. Siteman Cancer Center, Washington University School of Medicine, St Louis, Missouri

## Abstract

**Importance:**

Menopausal hormone therapy (MHT) is the treatment of choice for symptoms of menopause. However, its adoption is hindered by the risk-benefit trade-off in relation to acute and chronic diseases.

**Objective:**

To evaluate trends in and correlates of MHT use among postmenopausal women in the US from 1999 to March 2020.

**Design, Setting, and Participants:**

This serial cross-sectional analysis of MHT use used data from the nationally representative National Health and Nutrition Examination Survey (NHANES). Participants included noninstitutionalized US postmenopausal women from 10 NHANES study cycles (1999-2000 to 2017-March 2020 [pre–COVID-19 pandemic]). Data were analyzed from December 2023 to April 2024.

**Exposures:**

NHANES study cycle.

**Main Outcomes and Measures:**

Prevalence of MHT use was extracted from the prescription medication data collected during NHANES household interviews. MHT formulations were determined by hormone type.

**Results:**

Data on 13 048 US postmenopausal women (47.1% ≥65 years old) were analyzed. From 1999 to 2020, the prevalence of MHT use decreased among women of all age groups, from 26.9% (95% CI, 22.6%-31.7%) in 1999 to 4.7% (95% CI, 3.4%-6.5%) in 2020. Until 2002, MHT use was highest among women aged 52 to 65 years, but since 2005, MHT use has been highest among women younger than 52 years. MHT use decreased by 23.5% (95% CI, 11.4%-35.6%), 31.4% (95% CI, 23.4%-39.5%), and 10.6% (95% CI, 6.3%-14.8%) for women younger than 52 years, 52 years to younger than 65 years, and 65 years and older, respectively. Prevalence of MHT use decreased from 13.8% (95% CI, 8.5%-21.7%) to 2.6% (95% CI, 1.5%-4.6%) for Hispanic women, 11.9% (95% CI, 8.5%-16.3%) to 0.5% (95% CI, 0.2%-1.1%) for non-Hispanic Black women, and 31.4% (95% CI, 27.1%-36.1%) to 5.8% (95% CI, 4.1%-8.2%) for non-Hispanic White women. Non-Hispanic White women consistently had the highest prevalence of MHT use. Estrogen-only formulation accounted for more than 50% of the MHT for most study periods. The prevalence of MHT use varied by family income-to-poverty ratio, health insurance coverage in all racial and ethnic groups, weight, and smoking status among non-Hispanic White women, as well as by education attainment among non-Hispanic Black and Hispanic women.

**Conclusions and Relevance:**

Results of this cross-sectional study show that over the past 2 decades, MHT use declined among US postmenopausal women of all age and racial and ethnic groups. Women of racial and ethnic minority groups had lower prevalence of MHT use compared to non-Hispanic White women.

## Key Points

**Question:**

What were the prevalence and trends in menopausal hormone therapy (MHT) use among postmenopausal women in the US from 1999 through March 2020 (pre–COVID-19 pandemic)?

**Findings:**

In this serial cross-sectional study that included 13 048 postmenopausal women, the estimated prevalence of MHT use declined from 26.9% to 4.7% over 2 decades, with the greatest declines observed among women aged 52 years to younger than 65 years. Women of racial and ethnic minority groups had lower prevalence of MHT use compared to non-Hispanic White women.

**Meaning:**

MHT use declined among postmenopausal women in the US from 1999 to 2020, with considerable variation in patterns of use and correlates across age and racial and ethnic groups.

## Teaser

This cross-sectional study evaluates trends in and correlates of menopausal hormone therapy use among postmenopausal women in the US from 1999 to March 2020.

## Introduction

Annually, more than 1.3 million women in the US transition into menopause.^1^ Menopausal transition is associated with profound hormonal changes, which can be symptomatic in 80% of women, and these symptoms, including vasomotor, genitourinary, sleep difficulties, and cognition, can be severe in about 30% of women.^2^ Vasomotor symptoms, which are episodes of profuse heat accompanied by sweating and flushing, cause anxiety and sleep disruption and tend to be the most lifestyle-limiting symptom in many women.^3^

Menopausal hormone therapy (MHT) is the treatment of choice for menopausal symptoms and is the most effective treatment for vasomotor and genitourinary syndrome of menopause.^2,4^ During peak use in the late 1990s,^5^ prescriptions for MHT rose rapidly in the US, fueled by observational studies showing a protective effect on coronary heart diseases and cardiovascular deaths. MHT use, however, declined rapidly in 2002 after the Women’s Health Initiative trial showed that the health risks associated with MHT outweighed the benefits.^6,7^ Recent studies highlighting nuances in the risks of MHT with suggestions that the absolute risks of adverse events depend on several factors, including age at initiation and timing in relation to menopause, MHT type, dosage, duration, and route of administration, have led to several national societies putting out guidelines on MHT prescribing.^4,8,9^

In 2022, the US Preventive Services Task Force recommended against the use of MHT for primary prevention of chronic conditions in postmenopausal women,^10^ while the North American Menopause Society recommended tailoring MHT in treating menopausal symptoms to each individual need.^4^ The US Preventive Services Task Force also highlighted research gaps, including the need to understand the comparative benefits and harms of different MHT formulations and age at initiation. A gap in the current conversation on MHT use is an understanding of recent trends in, and prevalence of, MHT use. To our knowledge, the most recent study on MHT prevalence using a nationally representative sample was based on self-reported data up to 2010^7^; hence, there are no recent MHT use data captured by prescriptions to understand contemporary patterns in the US.

The goal of this cross-sectional study is to comprehensively describe the most recent trends in, and current prevalence and correlates of, MHT use, taking into consideration MHT formulations, among postmenopausal women in the US. Study findings will provide data that can help guide national discussions and policy on MHT use.

## Methods

### Study Population

The National Health and Nutrition Examination Survey (NHANES) was designed to provide cross-sectional estimates of the prevalence of health, nutrition, and potential risk factors among the civilian noninstitutionalized US population.^11^ The NHANES obtained approval from the National Center for Health Statistics Research Ethics Review Board, and participants provided written informed consent. The NHANES study has collected data in 2-year cycle intervals continuously since 1999 to 2000, with an interruption due to the COVID-19 pandemic in March 2020. In 2022, the 2017-2018 NHANES data were combined with data collected from 2019 to March 2020 to create the nationally representative 2017 to March 2020 prepandemic data. Data on prescription medications, menopausal status, sociodemographic characteristics, health insurance coverage, weight and height measures, and smoking status over 10 cycles from 1999 to 2000 to 2017 to March 2020 were extracted.^12^ This study followed the Strengthening the Reporting of Observational Studies in Epidemiology (STROBE) reporting guidelines for cross-sectional studies.

Menopausal status was derived from reproductive health questions (eTable 1 in Supplement 1).^13^ Women were asked if they had regular periods in the past 12 months (yes/no) and the reasons for not having regular periods (eg, pregnancy, breastfeeding, hysterectomy, menopause, other). Women were further asked if they had a hysterectomy (yes/no) and if they had both ovaries removed (yes/no). Women who self-reported menopause^14^ or had bilateral oophorectomy^15^ were included in the analyses. Those who had missing information on these questions were included in the analyses (5.7%) based on their age and smoking status (nonsmokers ≥52 years old; smokers ≥50 years old).^16^

### MHT

MHT use was extracted from the prescription medication data collected during household interviews. Participants were asked if any prescription medication was taken in the past month. Those who responded yes were asked to show containers of all of the products or verbally report the medication name when no container was available. Specific to MHT, 86% of the containers were seen. Each recorded medication was linked to the prescription drug database Lexicon Plus (Cerner Multum), which includes all prescription medications available in the US drug market to identify MHT.^17^

Female sex hormone medications were identified following the Lexicon Plus therapeutic classification scheme if the recorded medication met the following criteria: the first-level category was “hormones/hormone modifiers” and the second category was “sex hormone.” Authors reviewed the full list of identified female sex hormone medications and categorized them into contraceptives, MHT, contraceptive or MHT, or others (androgens and anabolic steroids, high-dose progestogen to prevent pregnancy loss, antineoplastics, and unspecified) based on the medication names and their third-level category on the Lexicon Plus therapeutic classification.

Medications that are specific MHT and medications that could function as either contraceptive or MHT taken among postmenopausal women were considered as MHT. The hormone type was further classified into estrogen only, progestogen only, combined estrogen and progestogen, combined estrogen and testosterone, and combined estrogen, progestogen, and testosterone (full list summarized in eTable 2 in Supplement 1).

### Correlates

Self-reported sociodemographic characteristics included age (<52, 52 to <65, and ≥65 years old), race and ethnicity (Hispanic, non-Hispanic Black, non-Hispanic White, and other, including multiracial), and educational attainment (<high school, high school, and >high school). Family income-to-poverty ratio (<1.3, 1.3 to <3.5, and ≥3.5) was calculated as the total family income divided by the poverty threshold with less than 1.3 defined as low-income status. Insurance coverage was self-reported (no health insurance, any health insurance, and private health insurance). Marital status was defined as living alone (widowed, divorced, separated, or never married) or living with someone (married or living with partner). Weight and height were measured during a physical examination in a mobile examination center or in the participant’s home following standard procedures. Body mass index (BMI) was calculated as weight in kilograms divided by height in meters squared and categorized into underweight or normal weight (<25), overweight (25.0-29.9), and obesity (≥30).^18^ Smoking status was classified into those who never smoked (did not smoke 100 cigarettes and do not smoke now), formerly smoked (smoked 100 cigarettes in life and do not smoke now), and currently smoke (smoked 100 cigarettes in life and smoke now).

### Statistical Analysis

Survey analysis procedures were used to account for the sample weights, stratification, and clustering of the complex sampling design to ensure nationally representative estimates.^19^ Sample sizes were estimated overall, by study cycle, and by correlates of MHT use. We calculated weighted prevalence and 95% CIs of MHT prevalence by race and ethnicity, age group, and NHANES study cycle. Logistic regression models were used to evaluate trends in MHT use from 1999 to March 2020 and estimated odds ratios (ORs) in crude and multivariable adjusted models using study cycles as an independent variable. Multivariable logistic regression models were adjusted for sociodemographic factors (age, race and ethnicity, family income-to-poverty ratio, educational attainment, health insurance coverage, marital status, BMI, and smoking status). Trends in MHT use over time were evaluated using study cycle as a continuous variable in univariate and multivariable regressions. Recent trends were evaluated by restricting data to between 2009 and 2010 and 2017 and March 2020. Hormone types were evaluated overall and restricted to women 52 years and older (ie, mean age at menopause for nonsmoking women in the US).^16^ Sensitivity analyses were conducted by (1) using the 2017-2018 NHANES as the most recent study cycle instead of the combined 2017 to March 2020 data, (2) excluding participants with a history of breast cancer, and (3) excluding participants with missing data on menopausal status or correlates.

Data were analyzed from December 2023 to April 2024. All statistical analyses were performed using Stata, version 17.0 (StataCorp). Statistical significance was set at *P* < .05. *P* values were not adjusted for multiple tests and should be interpreted as explanatory only.

## Results

Data on 13 048 US postmenopausal women from 10 NHANES cycles were analyzed. Participant sociodemographic and lifestyle behavior characteristics in the last cycle (2017-2020) are summarized in eTable 3 in Supplement 1. Most participants were non-Hispanic White (71.7%), had a family income-to-poverty ratio above 1.3 (1.3 to <3.5, 33.1%; ≥3.5, 40.5%), had more than high school education (59.6%), were enrolled in private insurance (61.4%), had overweight (26.8%) or obesity (40.6%), and never smoked (59.8%).

From 1999 to 2000 through 2017 to 2020, there was a statistically significant decline in the prevalence of MHT use among postmenopausal women overall, with a 22.2 (95% CI, 17.3-27.1) percentage point lower prevalence comparing the 1999-2000 cycle (26.9%; 95% CI, 22.6%-31.7%) with the 2017-2020 cycle (4.7%; 95% CI, 3.4%-6.5%) (*P* for trend <.001; Table 1 and eTable 4 in Supplement 1). MHT use decline was observed in all age groups (Figure, A), with the greatest absolute and relative declines observed among women aged 52 years to younger than 65 years (for age <52 years: difference, 23.5% [95% CI, 11.4%-35.6%]; prevalence ratio [PR], 0.27 [95% CI, 0.11-0.72]; for age 52 years to <65 years: difference, 31.4% [95% CI, 23.4%-39.5%]; PR, 0.12 [95% CI, 0.08-0.20]; and for age ≥65 years: difference, 10.6% [95% CI, 6.3%-14.8%]; PR, 0.29 [95% CI, 0.17-0.49]). Until 2002, prevalence of MHT use was highest among postmenopausal women aged 52 to 65 years but since 2005 has been highest among women younger than 52 years (Table 1). The estimated prevalence in 2017 to 2020 was 9.4% (95% CI, 3.9%-21.1%), 4.5% (95% CI, 2.9%-6.7%), and 4.3% (95% CI, 2.7%-6.7%) among postmenopausal women younger than 52 years, 52 years to younger than 65 years, and 65 years and older, respectively.

**Table 1.** Prevalence and Trends in Menopausal Hormone Therapy Use Among US Postmenopausal Women Overall and by Age Group^a^

Study cycle	Weighted prevalence, % (95% CI)			
	Overall	Age		
		<52 y	52 to <65 y	≥65 y
1999-2000	26.9 (22.6 to 31.7)	32.9 (25.0 to 42.0)	35.9 (28.7 to 43.9)	14.9 (11.5 to 18.9)
2001-2002	29.5 (25.2 to 34.2)	26.2 (18.7 to 35.5)	38.5 (31.9 to 45.5)	23.2 (18.7 to 28.4)
2003-2004	16.2 (13.9 to 18.9)	20.0 (14.1 to 27.5)	21.8 (18.3 to 25.8)	9.5 (6.5 to 13.6)
2005-2006	12.0 (10.1 to 14.2)	26.8 (17.6 to 38.5)	14.8 (10.7 to 20.1)	3.5 (2.2 to 5.5)
2007-2008	9.8 (7.6 to 12.5)	17.9 (12.6 to 24.7)	8.7 (6.0 to 12.4)	7.6 (4.9 to 11.5)
2009-2010	6.7 (5.0 to 8.9)	8.9 (4.7 to 16.1)	8.3 (5.4 to 12.5)	4.3 (2.5 to 7.3)
2011-2012	7.8 (6.0 to 10.1)	13.0 (8.6 to 19.1)	9.9 (6.4 to 15.1)	3.8 (1.9 to 7.2)
2013-2014	8.4 (6.4 to 10.9)	18.3 (12.2 to 26.5)	9.7 (6.7 to 13.8)	5.0 (3.4 to 7.3)
2015-2016	6.8 (4.1 to 11.2)	12.7 (5.9 to 25.5)	7.9 (4.1 to 14.5)	4.6 (2.6 to 8.0)
2017-2020	4.7 (3.4 to 6.5)	9.4 (3.9 to 21.1)	4.5 (2.9 to 6.7)	4.3 (2.7 to 6.7)
*P* value for trend	<.001	.05	<.001	.002
2017-2020 vs 1999-2000, difference (95% CI)^b^	−22.2 (−27.1 to −17.3)	−23.5 (−35.6 to −11.4)	−31.4 (−39.5 to −23.4)	−10.6 (−14.8 to −6.3)
2017-2020 vs 1999-2000, prevalence ratio (95% CI)^c^	0.18 (0.12 to 0.26)	0.27 (0.11 to 0.72)	0.12 (0.08 to 0.20)	0.29 (0.17 to 0.49)

^a^ All data are weighted to be nationally representative.

^b^ Indicates the absolute change in prevalence of menopausal hormone therapy use between 1999 and 2000 and 2017 and March 2020 (pre–COVID-19 pandemic).

^c^ Indicates the relative change in prevalence of menopausal hormone therapy use between 1999 and 2000 and 2017 and March 2020 (pre–COVID-19 pandemic).

**Figure.**
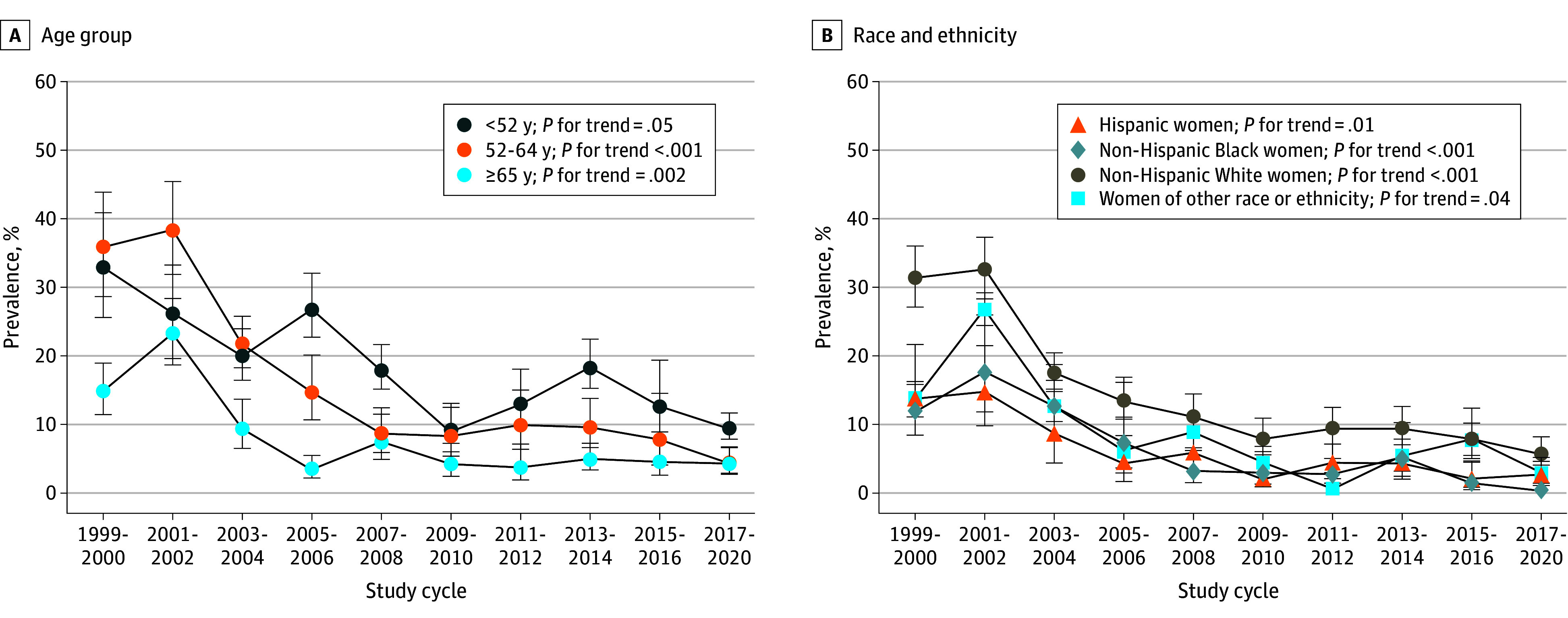
Crude Weighted Trends in Menopausal Hormone Therapy Use Among US Postmenopausal Women by Age Group and Race and Ethnicity Trends in menopausal hormone therapy use over time were evaluated using *P* values for trend by modeling study cycle as a continuous variable in univariate regression models. Data are from the National Health and Nutrition Examination Survey, 1999 to March 2020 (pre–COVID-19 pandemic), and were weighted to be nationally representative. Error bars indicate 95% CIs.

The prevalence of MHT use declined over the 20-year period in each racial and ethnic group (Table 2 and Figure, B). The estimated prevalence declined from 13.8% (95% CI, 8.5%-21.7%) to 2.6% (95% CI, 1.5%-4.6%) for Hispanic women, 11.9% (95% CI, 8.5%-16.3%) to 0.5% (95% CI, 0.2%-1.1%) for non-Hispanic Black women, 31.4% (95% CI, 27.1%-36.1%) to 5.8% (95% CI, 4.1%-8.2%) for non-Hispanic White women, and 13.5% (95% CI, 3.3%-41.5%) to 2.8% (95% CI, 1.1%-7.1%) for women of other racial or ethnic groups. Non-Hispanic Black women had the lowest prevalence of MHT use in 1999 to 2000 and 2017 to 2020 and exhibited the greatest relative decline (PR, 0.04; 95% CI, 0.02-1.00). Although the prevalence of MHT use was highest for non-Hispanic White women at all time points compared to women of other racial or ethnic groups, the relative declines in prevalence were similar between Hispanic women (PR, 0.19; 95% CI, 0.09-0.41), non-Hispanic White women (PR, 0.18; 95% CI, 0.12-0.27), and women of other racial or ethnic groups (PR, 0.21; 95% CI, 0.04-1.09).

**Table 2.** Prevalence and Trends in Menopausal Hormone Therapy Use Among US Postmenopausal Women by Race and Ethnicity^a^

Study cycle	Weighted prevalence, % (95% CI)			
	Hispanic	Non-Hispanic Black	Non-Hispanic White	Other race or ethnicity^b^
1999-2000	13.8 (8.5 to 21.7)	11.9 (8.5 to 16.3)	31.4 (27.1 to 36.1)	13.5 (3.3 to 41.5)
2001-2002	14.8 (9.9 to 21.5)	17.8 (11.8 to 26.0)	32.7 (28.3 to 37.3)	26.8 (12.7 to 48.2)
2003-2004	8.7 (4.4 to 16.4)	12.7 (8.4 to 18.8)	17.5 (14.8 to 20.5)	12.8 (4.8 to 30.2)
2005-2006	4.4 (1.7 to 10.8)	7.3 (3.0 to 16.9)	13.4 (11.1 to 16.2)	6.1 (1.2 to 25.3)
2007-2008	6.0 (3.9 to 9.0)	3.2 (1.6 to 6.3)	11.2 (8.6 to 14.5)	9.0 (2.2 to 30.8)
2009-2010	2.0 (0.9 to 4.4)	2.9 (1.3 to 6.1)	7.9 (5.6 to 11.0)	4.5 (0.9 to 18.9)
2011-2012	4.5 (2.1 to 9.3)	2.7 (1.4 to 5.0)	9.5 (7.1 to 12.5)	0.6 (0.1 to 3.8)
2013-2014	4.4 (2.0 to 9.4)	5.1 (2.5 to 10.3)	9.5 (7.1 to 12.7)	5.5 (2.7 to 10.9)
2015-2016	2.0 (0.9 to 4.7)	1.5 (0.5 to 4.5)	8.0 (5.0 to 12.4)	7.9 (1.5 to 32.4)
2017-2020	2.6 (1.5 to 4.6)	0.5 (0.2 to 1.1)	5.8 (4.1 to 8.2)	2.8 (1.1 to 7.1)
*P* value for trend	.005	.001	<.001	.04
2017-2020 vs 1999-2000, difference (95% CI)^c^	−11.2 (−18.0 to −4.3)	−11.4 (−15.4 to −7.4)	−25.6 (−30.7 to −20.6)	−10.7 (−29.0 to 7.7)
2017-2020 vs 1999-2000, prevalence ratio (95% CI)^d^	0.19 (0.09 to 0.41)	0.04 (0.02 to 0.10)	0.18 (0.12 to 0.27)	0.21 (0.04 to 1.09)

^a^ All data are weighted to be nationally representative.

^b^ Participants self-selected into the other race or ethnicity category, which includes race and ethnicity other than non-Hispanic white, non-Hispanic black, or Hispanic, including multiracial.

^c^ Indicates the absolute change in prevalence of menopausal hormone therapy use between 1999 and 2000 and 2017 and March 2020 (pre–COVID-19 pandemic).

^d^ Indicates the relative change in prevalence of menopausal hormone therapy use between 1999 and 2000 and 2017 and March 2020 (pre–COVID-19 pandemic).

Of prescribed MHT during the 2017-2020 cycle, estrogen-only formulations accounted for 52.8% (95% CI, 37.2%-67.9%), progestogen-only formulations for 10.5% (95% CI, 3.4%-27.9%), combined estrogen and progestogen for 36.1% (95% CI, 19.2%-57.3%), and combined estrogen and testosterone for 0.6% (95% CI, 0.1%-4.3%) (Table 3). Except for the 2015-2016 cycle where it accounted for 38.2% (95% CI, 17.3%-64.5%), estrogen-only formulations accounted for more than 50% of MHT use at every study cycle, being as high as 78.0% (95% CI, 69.2%-84.9%) in 2007 to 2008. There were drastic switches from using progestogen-only and combined estrogen and progestogen formulations to estrogen-only formulations after 2001 to 2002, but this trend reversed after the 2007-2008 cycle. These estimates remained similar when restricted to women 52 years and older (eTable 5 in Supplement 1).

**Table 3.** Menopausal Hormone Therapy Formulation Use Among US Postmenopausal Women^a^

Study cycle	Weighted prevalence, % (95% CI)				
	Estrogen only	Progestogen only	Estrogen + progestogen	Estrogen + testosterone	Estrogen + progestogen + testosterone
1999-2000	58.3 (49.8-66.3)	3.3 (1.0-10.2)	28.6 (22.2-36.1)	7.6 (3.9-14.3)	2.2 (0.7-6.2)
2001-2002	62.0 (54.6-68.8)	1.4 (0.5-4.2)	32.3 (25.1-40.3)	3.6 (2.0-6.5)	0.7 (0.2-3.0)
2003-2004	73.4 (65.2-80.3)	0	20.8 (14.9-28.4)	5.7 (2.9-11.1)	0
2005-2006	74.2 (59.3-85.0)	0	18.3 (10.5-29.9)	5.4 (1.7-15.7)	2.1 (0.3-12.9)
2007-2008	78.0 (69.2-84.9)	0.5 (0.1-3.8)	18.2 (11.0-28.5)	3.3 (1.1-9.6)	0
2009-2010	57.9 (46.1-69.0)	3.6 (0.9-13.5)	33.7 (22.8-46.8)	4.7 (0.9-21.7)	0
2011-2012	65.5 (48.2-79.5)	4.0 (0.8-18.2)	28.6 (15.4-46.9)	1.9 (0.2-12.7)	0
2013-2014	55.2 (43.4-66.5)	8.1 (4.4-14.6)	22.0 (11.7-37.6)	11.1 (4.0-27.3)	3.5 (0.5-20.5)
2015-2016	38.2 (17.3-64.5)	25.0 (4.7-69.1)	36.8 (13.2-69.0)	0	0
2017-2020	52.8 (37.2-67.9)	10.5 (3.4-27.9)	36.1 (19.2-57.3)	0.6 (0.1-4.3)	0

^a^ All data are weighted to be nationally representative.

Table 4 presents multivariable analyses that are adjusted for correlates overall and by racial and ethnic groups. Overall, the prevalence of MHT use decreased with age (OR, 0.96; 95% CI, 0.95-0.97). Compared to non-Hispanic White women, non-Hispanic Black women (OR, 0.44; 95% CI, 0.35-0.57) and Hispanic women (OR, 0.46; 95% CI, 0.35-0.60) as well as women of other racial or ethnic groups (OR, 0.55; 95% CI, 0.34-0.85) had lower prevalence of MHT use. Additionally, higher prevalence of MHT use was associated with higher family income-to-poverty ratio (1.3 to <3.5 vs <1.3: OR, 1.57; 95% CI, 1.23-2.01; ≥3.5 vs <1.3: OR, 2.14; 95% CI, 1.65-2.79) and health insurance coverage (any insurance vs no insurance: OR, 2.06; 95% CI, 1.37-3.10; private insurance vs no insurance: OR, 1.97; 95% CI, 1.37-2.84). Educational attainment (>high school vs <high school) was associated with MHT use in non-Hispanic Black women (OR, 3.47; 95% CI, 2.13-5.63) and Hispanic women (OR, 1.79; 95% CI, 1.09-2.95) but not among non-Hispanic White women (OR, 1.01; 95% CI, 0.77-1.32). Having a BMI of 30 or greater was associated with lower prevalence of MHT use (OR, 0.64; 95% CI, 0.50-0.81) among non-Hispanic White women. Currently smoking was inversely associated with MHT use in non-Hispanic White women (OR, 0.75; 95% CI, 0.58-0.98) but positively associated with use in Hispanic women (OR, 1.95; 95% CI, 1.05-3.63). No association was observed between MHT use and marital status.

**Table 4.** Weighted Logistic Regression of Menopausal Hormone Therapy Use Among US Postmenopausal Women by Race and Ethnicity^a^

Variable	Odds ratio (95% CI)^b^				
	Overall	Hispanic	Non-Hispanic Black	Non-Hispanic White	Other^c^
Age	0.96 (0.95-0.97)	0.98 (0.95-1.00)	0.98 (0.96-1.00)	0.96 (0.95-0.97)	0.94 (0.89-1.00)
Race and ethnicity					
Hispanic	0.46 (0.35-0.60)	NA	NA	NA	NA
Non-Hispanic Black	0.44 (0.35-0.57)	NA	NA	NA	NA
Non-Hispanic White	1 [Reference]	NA	NA	NA	NA
Other^c^	0.54 (0.34-0.85)	NA	NA	NA	NA
Family income-to-poverty ratio					
<1.3	1 [Reference]	1 [Reference]	1 [Reference]	1 [Reference]	1 [Reference]
1.3 to <3.5	1.57 (1.23-2.01)	1.18 (0.66-2.10)	1.51 (0.82-2.78)	1.56 (1.16-2.10)	3.42 (0.86-13.64)
≥3.5	2.14 (1.65-2.79)	2.16 (1.16-4.03)	1.86 (1.10-3.12)	2.07 (1.51-2.83)	7.35 (1.30-41.40)
Missing	1.13 (0.77-1.65)	0.88 (0.40-1.96)	1.41 (0.49-4.06)	1.12 (0.72-1.75)	1.29 (0.11-14.64)
Educational attainment					
<High school	1 [Reference]	1 [Reference]	1 [Reference]	1 [Reference]	1 [Reference]
High school	1.02 (0.80-1.30)	0.70 (0.38-1.30)	2.50 (1.36-4.58)	0.89 (0.65-1.20)	2.32 (0.53-10.18)
>High school	1.19 (0.95-1.48)	1.79 (1.09-2.95)	3.47 (2.13-5.63)	1.01 (0.77-1.32)	2.01 (0.50-8.15)
Health insurance					
No insurance	1 [Reference]	1 [Reference]	1 [Reference]	1 [Reference]	1 [Reference]
Any insurance	2.06 (1.37-3.10)	3.09 (1.15-8.32)	3.58 (1.49-8.61)	1.75 (1.10-2.78)	1.63 (0.54-4.94)
Private insurance	1.97 (1.37-2.84)	3.85 (1.68-8.83)	3.44 (1.41-8.41)	1.66 (1.10-2.51)	NA
Missing	0.96 (0.27-3.50)	1.82 (0.32-10.43)	2.25 (0.20-25.77)	0.85 (0.10-7.08)	NA
Marital status					
Living alone	1 [Reference]	1 [Reference]	1 [Reference]	1 [Reference]	1 [Reference]
Living with someone	1.11 (0.93-1.34)	1.11 (0.72-1.72)	1.55 (1.04-2.32)	1.12 (0.92-1.36)	0.46 (0.17-1.26)
Missing	0.46 (0.27-0.81)	2.14 (0.29-15.85)	0.50 (0.07-3.57)	0.34 (0.17-0.69)	5.13 (0.33-79.65)
BMI					
<25	1 [Reference]	1 [Reference]	1 [Reference]	1 [Reference]	1 [Reference]
25 to <30	1.00 (0.81-1.24)	1.60 (0.88-2.92)	1.13 (0.66-1.94)	0.94 (0.74-1.19)	2.16 (0.70-6.61)
≥30	0.68 (0.56-0.84)	0.90 (0.48-1.71)	0.83 (0.49-1.40)	0.64 (0.50-0.81)	1.81 (0.56-5.81)
Missing	0.65 (0.47-0.90)	1.26 (0.43-3.72)	0.43 (0.15-1.23)	0.62 (0.44-0.89)	0.95 (0.17-5.30)
Smoking status					
Never	1 [Reference]	1 [Reference]	1 [Reference]	1 [Reference]	1 [Reference]
Former	1.15 (0.96-1.39)	1.21 (0.78-1.87)	1.29 (0.82-2.04)	1.11 (0.90-1.37)	1.76 (0.70-4.40)
Current	0.85 (0.67-1.08)	1.95 (1.05-3.63)	1.15 (0.68-1.92)	0.75 (0.58-0.98)	0.67 (0.22-2.06)
Missing	1.44 (0.16-12.61)	7.80 (1.13-53.77)	7.80 (1.13-53.77)	1.42 (0.12-16.61)	NA
Study cycle					
1999-2000	1 [Reference]	1 [Reference]	1 [Reference]	1 [Reference]	1 [Reference]
2001-2002	0.95 (0.68-1.33)	0.97 (0.41-2.28)	1.27 (0.59-2.73)	0.91 (0.64-1.28)	2.14 (0.22-20.54)
2003-2004	0.43 (0.33-0.57)	0.57 (0.19-1.78)	0.75 (0.38-1.47)	0.40 (0.30-0.54)	0.94 (0.10-8.77)
2005-2006	0.29 (0.21-0.39)	0.27 (0.07-1.07)	0.39 (0.14-1.06)	0.28 (0.20-0.38)	0.43 (0.03-5.64)
2007-2008	0.23 (0.17-0.33)	0.37 (0.15-0.91)	0.17 (0.06-0.43)	0.22 (0.15-0.32)	0.62 (0.06-6.00)
2009-2010	0.15 (0.10-0.23)	0.14 (0.05-0.40)	0.14 (0.06-0.36)	0.15 (0.10-0.23)	0.24 (0.02-2.75)
2011-2012	0.18 (0.13-0.27)	0.34 (0.11-1.06)	0.14 (0.07-0.32)	0.19 (0.13-0.27)	0.05 (0.00-0.72)
2013-2014	0.21 (0.14-0.32)	0.25 (0.10-0.64)	0.29 (0.11-0.74)	0.20 (0.13-0.31)	0.48 (0.05-4.28)
2015-2016	0.16 (0.09-0.27)	0.13 (0.04-0.43)	0.08 (0.02-0.28)	0.15 (0.09-0.26)	0.75 (0.06-9.04)
2017-2020	0.12 (0.08-0.18)	0.14 (0.05-0.34)	0.03 (0.01-0.07)	0.12 (0.08-0.18)	0.16 (0.02-1.31)
*P* value for trend^d^	<.001	.002	.002	<.001	.01

Abbreviations: BMI, body mass index (calculated as weight in kilograms divided by height in meters squared); NA, not applicable.

^a^ All data are weighted to be nationally representative.

^b^ Odds ratios represent the change in odds expected in each category compared with the reference group.

^c^ Participants self-selected into the other race or ethnicity category, which includes race and ethnicity other than non-Hispanic white, non-Hispanic black, or Hispanic, including multiracial.

^d^ *P* value for trend over the study cycle was calculated using the National Health and Nutrition Examination Survey study 2-year survey cycle as a continuous variable.

Recent MHT use in the past decade (2009-2010 to 2017-March 2020) continued to decline among postmenopausal women. Nevertheless, this continued decline in MHT use was observed among women aged 52 years to younger than 65 years but not in other age groups (eTable 6 in Supplement 1). Furthermore, the continued decline was observed among non-Hispanic White women and non-Hispanic Black women but not Hispanic women and women of other racial or ethnic groups (eTable 6 in Supplement 1). Estimates from sensitivity analyses were consistent when using 2017 to 2018 as the most recent cycle (see eTable 7 in Supplement 1 for sample characteristics, eTable 8 in Supplement 1 for hormone type, eTable 9 in Supplement 1 for prevalence and trends, and eTable 10 in Supplement 1 for multivariable regressions), excluding participants with a history of breast cancer diagnosis (see eTable 8 in Supplement 1 for hormone type, eTable 9 in Supplement 1 for prevalence and trends, and eTable 10 in Supplement 1 for multivariable regressions), and excluding participants with missing data on menopausal status or correlates (see eTable 4 in Supplement 1 for sample size, eTable 8 in Supplement 1 for hormone type, eTable 9 in Supplement 1 for prevalence and trends, and eTable 10 in Supplement 1 for multivariable regressions).

## Discussion

In this nationally representative sample of US postmenopausal women, we observed a continued decline in MHT use among women in all included racial and ethnic groups over the 20-year period. In sharp contrast to a high of 26.9% in 1999 to 2000, the contemporary prevalence of MHT use was 4.7%. Marked differences in trends, current prevalence, and correlates of MHT use were observed across age and racial and ethnic groups. Compared to non-Hispanic White women, non-Hispanic Black and Hispanic women had consistently lower prevalence of MHT use. Estrogen-only formulations accounted for the majority of prescribed MHT, followed by combined estrogen and progestogen, with fewer women taking progestogen-only and combined estrogen and testosterone formulations.

These results support findings from earlier studies in the US that showed a decrease in MHT use after 2002.^7,20,21^ The most recent of these studies analyzed data collected up to 2013 and reported decreases in MHT initiation across all racial and ethnic groups.^21^ The decrease was highest for non-Hispanic Black women, those with less than high school education, and those older than 57 years. Studies in other countries such as Australia, Germany, Korea, and the UK have reported similar declines in the prevalence of MHT use,^22,23,24,25^ but most of those studies with the exception of the UK study were limited to trends up until 2016. Although the UK study also reported a decrease in the annual prescribing prevalence from 7.9 of 100 women in 2010 to 6.9 of 100 women in 2020, there was a 13.6% increase in the number of women receiving their first MHT prescription.^26^ This increase was observed across all age groups and for all MHT formulations, but the largest absolute increases were among younger postmenopausal women aged 50 to 59 years for transdermal administrations and combined estrogen and progestogen formulations.^26^ In contrast, the present findings in the US context indicated that MHT use continued to decline among US women from 2009 to 2020, particularly among women aged 52 to 65 years.

MHT remains the most effective treatment for menopausal symptoms, and professional societies in North America recommend it primarily for managing vasomotor symptoms, genitourinary syndromes, and bone loss arising from menopause, though not for the prevention of coronary heart diseases or any other chronic conditions.^4^ They also recommend starting MHT as early as possible before 60 years of age when the risk-benefit ratio is favorable, as well as using the lowest effective dose for the shortest duration necessary to manage symptoms and tailoring MHT use to each woman with a periodic risk-benefit reassessment.

The present study showed that although estrogen-only formulations are still the most widely prescribed MHT, the use of combined estrogen plus progestogen is gaining ground once more, accounting for more than one-third of current MHT use. MHT formulations are associated with different risk profiles, with a better safety profile for estrogen only. Long-term follow-up from Women’s Health Initiative trials showed an elevated risk of breast cancer among women with a uterus assigned to conjugated equine estrogen plus medroxyprogesterone acetate, but a reduced risk among women with prior hysterectomy assigned to conjugated equine estrogen alone.^27^

Although menopausal symptoms affect women of all races and ethnicities, racial differences in menopausal transition have been documented, with non-Hispanic Black women having an earlier start and longer duration of transition than non-Hispanic White women.^14^ Additionally, non-Hispanic Black women are more likely to experience severe menopausal symptoms but less likely to receive treatment for these symptoms.^28^ These symptoms not only considerably impair women’s quality of life, but also lead to an estimated $1.8 billion in annual cost due to adverse outcomes in the US.^29^ Studies are needed to determine which of the current MHTs are safest and most acceptable for use among women with symptomatic menopausal transition.

### Strengths and Limitations

A strength of this study is the comprehensive description of the most recent trends, current prevalence, and correlates of MHT use among US postmenopausal women using 20 years of nationally representative data. MHT use was determined during in-home interviews conducted among NHANES participants. Furthermore, up to 86% of the prescription containers were seen by interviewers, ensuring accurate data on MHT use.

This study also has several limitations. First, the route of administration for MHT was not documented in NHANES; hence, it was not evaluated in the present analyses. Second, hormone therapy is routine in women who have bilateral oophorectomy prior to the final menstrual period. Insufficient information was available to assess whether bilateral oophorectomy occurred prior to the last menstrual period in the analyzed sample. Nevertheless, the proportion of women who were younger than the mean age at menopause was small (6.6%). Third, the reasons for using MHT were not fully documented in NHANES. Therefore, medications that could function as both contraceptive and MHT taken by perimenopausal women for contraceptive may have been classified as MHT in the present analyses. Nonetheless this misclassification should be minimal and would not have affected analyses evaluating trends.

## Conclusions

In this serial cross-sectional study using nationally representative data from 1999 through March 2020 (pre–COVID-19 pandemic), we report continued declines in MHT use across women of all ages and racial and ethnic groups. Women of racial and ethnic minority groups had lower prevalence of MHT use compared to non-Hispanic White women. Discussions and policy on MHT use need to take into consideration sociodemographic factors such as age, race and ethnicity, income, and education.

## Notes

Supplement 1. **eTable 1.** Variables to ascertain menopause status in NHANES study cycle, 1999-2000 to 2017-March 2020 pre-pandemic **eTable 2.** Menopausal hormone therapy included in the analyses by Multum Lexicon Therapeutic Classification Scheme **eTable 3.** Sociodemographic characteristics of postmenopausal women from the NHANES overall and by age group and race/ethnicity, 2017-March 2020 pre-pandemic **eTable 4.** Sample size of postmenopausal women from the NHANES by study cycle and with complete data on correlates by study cycle, 1999-March 2020 pre-pandemic **eTable 5.** Menopausal hormone therapy formulations prescribed among US postmenopausal women ≥52 years 1999-March 2020 pre-pandemic **eTable 6.** Prevalence and trends in menopausal hormone therapy use among US postmenopausal women overall and by age group and race/ethnicity, 2009-March 2020 pre-pandemic **eTable 7.** Sociodemographic characteristics of postmenopausal women from the NHANES, 2017-2018 **eTable 8.** Menopausal hormone therapy formulations prescribed among US postmenopausal women, 1999-2018, postmenopausal women with no history of breast cancer, 1999-March 2020 pre-pandemic, and postmenopausal women with complete data on corelates, 1999-March 2020 pre-pandemic **eTable 9.** Prevalence and trends in menopausal hormone therapy use among US postmenopausal women overall and by age group, 1999-2018, postmenopausal women by race/ethnicity, 1999-2018, postmenopausal women with no history of breast cancer overall and by age group, 1999-March 2020 pre-pandemic, postmenopausal women with no history of breast cancer by race/ethnicity, 1999-March 2020 pre-pandemic, postmenopausal women with complete data on correlates overall and by age group, 1999-March 2020 pre-pandemic, and postmenopausal women with complete data on correlates by race/ethnicity, 1999-March 2020 pre-pandemic **eTable 10.** Weighted logistic regression of menopausal hormone therapy use among US postmenopausal women by race/ethnicity, 1999-2018, postmenopausal women with no history of breast cancer, 1999-March 2020 pre-pandemic, and postmenopausal women with complete data on correlates, 1999-March 2020 pre-pandemic

Supplement 2. Data Sharing Statement
