# Supplementary material for: Menopausal Hormone Therapy Use Among Postmenopausal Women
Source: JAMA Health Forum. 2024 Sep 27;5(9):e243128. doi: 10.1001/jamahealthforum.2024.3128 (PMC11437377; doi:10.1001/jamahealthforum.2024.3128)
Supplement: Supplement 1. — eTable 1. Variables to ascertain menopause status in NHANES study cycle, 1999-2000 to 2017-March 2020 pre-pandemic eTable 2. Menopausal hormone therapy included in the analyses by Multum Lexicon Therapeutic Classification Scheme eTable 3. Sociodemographic characteristics of postmenopausal women from the NHANES overall and by age group and race/ethnicity, 2017-March 2020 pre-pandemic eTable 4. Sample size of postmenopausal women from the NHANES by study cycle and with complete data on correlates by study cycle, 1999-March 2020 pre-pandemic eTable 5. Menopausal hormone therapy formulations prescribed among US postmenopausal women ≥52 years 1999-March 2020 pre-pandemic eTable 6. Prevalence and trends in menopausal hormone therapy use among US postmenopausal women overall and by age group and race/ethnicity, 2009-March 2020 pre-pandemic eTable 7. Sociodemographic characteristics of postmenopausal women from the NHANES, 2017-2018 eTable 8. Menopausal hormone therapy formulations prescribed among US postmenopausal women, 1999-2018, postmenopausal women with no history of breast cancer, 1999-March 2020 pre-pandemic, and postmenopausal women with complete data on corelates, 1999-March 2020 pre-pandemic eTable 9. Prevalence and trends in menopausal hormone therapy use among US postmenopausal women overall and by age group, 1999-2018, postmenopausal women by race/ethnicity, 1999-2018, postmenopausal women with no history of breast cancer overall and by age group, 1999-March 2020 pre-pandemic, postmenopausal women with no history of breast cancer by race/ethnicity, 1999-March 2020 pre-pandemic, postmenopausal women with complete data on correlates overall and by age group, 1999-March 2020 pre-pandemic, and postmenopausal women with complete data on correlates by race/ethnicity, 1999-March 2020 pre-pandemic eTable 10. Weighted logistic regression of menopausal hormone therapy use among US postmenopausal women by race/ethnicity, 1999-2018, postmenopausal women with no history of [file jamahealthforum-e243128-s001.pdf]

Supplemental Online Content

Yang L, Toriola AT. Menopausal hormone therapy use among postmenopausal women. *JAMA Health Forum*. 2024;5(9):e243128. doi:10.1001/jamahealthforum.2024.3128

eTable 1. Variables to ascertain menopause status in NHANES study cycle, 1999-2000 to 2017-March 2020 pre-pandemic
eTable 2. Menopausal hormone therapy included in the analyses by Multum Lexicon Therapeutic Classification Scheme
eTable 3. Sociodemographic characteristics of postmenopausal women from the NHANES overall and by age group and race/ethnicity, 2017-March 2020 pre-pandemic
eTable 4. Sample size of postmenopausal women from the NHANES by study cycle and with complete data on correlates by study cycle, 1999-March 2020 pre-pandemic
eTable 5. Menopausal hormone therapy formulations prescribed among US postmenopausal women ≥52 years 1999-March 2020 pre-pandemic
eTable 6. Prevalence and trends in menopausal hormone therapy use among US postmenopausal women overall and by age group and race/ethnicity, 2009-March 2020 pre-pandemic
eTable 7. Sociodemographic characteristics of postmenopausal women from the NHANES, 2017-2018
eTable 8. Menopausal hormone therapy formulations prescribed among US postmenopausal women, 1999-2018, postmenopausal women with no history of breast cancer, 1999-March 2020 pre-pandemic, and postmenopausal women with complete data on corelates, 1999-March 2020 pre-pandemic
eTable 9. Prevalence and trends in menopausal hormone therapy use among US postmenopausal women overall and by age group, 1999-2018, postmenopausal women by race/ethnicity, 1999-2018, postmenopausal women with no history of breast cancer overall and by age group, 1999-March 2020 pre-pandemic, postmenopausal women with no history of breast cancer by race/ethnicity, 1999-March 2020 pre-pandemic, postmenopausal women with complete data on correlates overall and by age group, 1999-March 2020 pre-pandemic, and postmenopausal women with complete data on correlates by race/ethnicity, 1999-March 2020 pre-pandemic
eTable 10. Weighted logistic regression of menopausal hormone therapy use among US postmenopausal women by race/ethnicity, 1999-2018, postmenopausal women with no history of breast cancer, 1999-March 2020 pre-pandemic, and postmenopausal women with complete data on correlates, 1999-March 2020 pre-pandemic

This supplemental material has been provided by the authors to give readers additional information about their work.

© 2024 Yang and Toriola. *JAMA Health Forum.*

eTable 1. Variables to ascertain menopause status in NHANES study cycle, 1999-2000 to 2017-March 2020 pre-pandemic		
Study cycle	Variable	English Text
1999-2000	rhq030	{Have you/Has SP} had regular periods in the past 12 months? {Please do not include bleedings caused by medical conditions or surgeries.}<br>(1: yes, 2: No, 7: Refused; 9: Don't know, ".": Missing)
	rhq040	What is the reason that {you have/SP has} not had regular periods in the past 12 months?<br>(1: Pregnant now; 2: Breast feeding; 3: Pregnant in past year; 4: Periods usually irregular; 5: Going-gone through menopause; 6: Medical conditions-treatments; 77: Refused; 99: Don't know; "." Missing)
	rhq310	Were both ovaries removed or only one?<br>(1: Both; 2: One; 7: Refused; 9: Don't know; "." Missing)
2001-2002	rhq030	{Have you/Has SP} had regular periods in the past 12 months? {Please do not include bleedings caused by medical conditions or surgeries.}<br>(1: yes, 2: No, 7: Refused; 9: Don't know, ".": Missing)
	rhq040	What is the reason that {you have/SP has} not had regular periods in the past 12 months?<br>(1: Pregnant now; 2: Breast feeding; 3: Pregnant in past year; 4: Periods usually irregular; 5: Going-gone through menopause; 6: Medical conditions-treatments; 77: Refused; 99: Don't know; "." Missing)
	rhq310	Were both ovaries removed or only one?<br>(1: Both; 2: One; 7: Refused; 9: Don't know; "." Missing)
2003-2004	rhq031	{Have you/Has SP} had at least one menstrual period in the past 12 months? (Please do not include bleedings caused by medical conditions, hormone therapy, or surgeries.)<br>(1: Yes; 2: No; 7: Refused; 9: Don't know; ".'" Missing)
	rhd042	What is the reason that {you have/SP has} not had a period in the past 12 months?<br>(1: Pregnancy; 2: Breastfeeding; 7: Menopause/Hysterectomy; 8: Medical conditions/treatments; 9: Other; 77: Refused; 99: Don't know; ".": Missing)
	rhq310	Were both ovaries removed or only one?<br>(1: Both; 2: One; 7: Refused; 9: Don't know' "." Missing)
2005-2006	rhq031	{Have you/Has SP} had at least one menstrual period in the past 12 months?<br>(Please do not include bleedings caused by medical conditions, hormone therapy, or surgeries.)<br>(1: Yes; 2: No; 7: Refused; 9: Don't know; ".'" Missing)
	rhd042	What is the reason that {you have/SP has} not had a period in the past 12 months?<br>(1: Pregnancy; 2: Breast feeding; 7: Menopause/Hysterectomy; 8: Medical conditions/treatments; 9: Other; 77: Refused; 99: Don't know; ".": Missing)
	rhq310	Were both ovaries removed or only one? (1: Both; 2: One; 7: Refused; 9: Don't know' "." Missing)
2007-2008	rhq031	{Have you/Has SP} had at least one menstrual period in the past 12 months? (Please do not include bleedings caused by medical conditions, hormone therapy, or surgeries.)<br>(1: yes, 2: No, 7: Refused; 9: Don't know, ".": Missing)
	rhd042	What is the reason that {you have/SP has} not had a period in the past 12 months?<br>(1: Pregnancy; 2: Breast feeding; 7: Menopause/Hysterectomy; 8: Medical conditions/treatments; 9: Other; 77: Refused; 99: Don't know; ".": Missing)

© 2024 Yang and Toriola. *JAMA Health Forum.*

	rhq305	{Have you/Has SP} had both of {your/her} ovaries removed (either when {you/she} had {your/her} uterus removed or at another time)?<br>(1: Yes; 2: No; 7 Refused; 9: Don't know; "." Missing)
2009-2010	rhq031	{Have you/Has SP} had at least one menstrual period in the past 12 months? (Please do not include bleedings caused by medical conditions, hormone therapy, or surgeries.)<br>(1: yes, 2: No, 7: Refused; 9: Don't know, ".": Missing)
	rhd042	What is the reason that {you have/SP has} not had a period in the past 12 months?<br>(1: Pregnancy; 2: Breast feeding; 7: Menopause/Hysterectomy; 8: Medical conditions/treatments; 9: Other; 77: Refused; 99: Don't know; ".": Missing)
	rhq305	{Have you/Has SP} had both of {your/her} ovaries removed (either when {you/she} had {your/her} uterus removed or at another time)?<br>(1: Yes; 2: No; 7 Refused; 9: Don't know; "." Missing)
2011-2012	rhq031	{Have you/Has SP} had at least one menstrual period in the past 12 months? (Please do not include bleedings caused by medical conditions, hormone therapy, or surgeries.)<br>(1: yes, 2: No, 7: Refused; 9: Don't know, ".": Missing)
	rhd042	What is the reason that {you have/SP has} not had a period in the past 12 months?<br>(1: Pregnancy; 2: Breast feeding; 7: Menopause/Hysterectomy; 8: Medical conditions/treatments; 9: Other; 77: Refused; 99: Don't know; ".": Missing)
	rhq305	{Have you/Has SP} had both of {your/her} ovaries removed (either when {you/she} had {your/her} uterus removed or at another time)?<br>(1: Yes; 2: No; 7 Refused; 9: Don't know; "." Missing)
2013-2014	rhq031	{Have you/Has SP} had at least one menstrual period in the past 12 months? (Please do not include bleedings caused by medical conditions, hormone therapy, or surgeries.)<br>(1: yes, 2: No, 7: Refused; 9: Don't know, ".": Missing)
	rhd043	What is the reason that {you have/SP has} not had a period in the past 12 months?<br>(1: Pregnancy; 2: Breast feeding; 3: Hysterectomy; 7: Menopause/Change of life; 9: Other; 9: Other; 77: Refused; 99: Don't know; ".": Missing)
	rhq305	{Have you/Has SP} had both of {your/her} ovaries removed (either when {you/she} had {your/her} uterus removed or at another time)?<br>(1: Yes; 2: No; 7 Refused; 9: Don't know; "." Missing)
2015-2016	rhq031	{Have you/Has SP} had at least one menstrual period in the past 12 months? (Please do not include bleedings caused by medical conditions, hormone therapy, or surgeries.)<br>(1: yes, 2: No, 7: Refused; 9: Don't know, ".": Missing)
	rhd043	What is the reason that {you have/SP has} not had a period in the past 12 months?<br>(1: Pregnancy; 2: Breast feeding; 3: Hysterectomy; 7: Menopause/Change of life; 9: Other; 9: Other; 77: Refused; 99: Don't know; ".": Missing)
	rhq305	{Have you/Has SP} had both of {your/her} ovaries removed (either when {you/she} had {your/her} uterus removed or at another time)?<br>(1: Yes; 2: No; 7 Refused; 9: Don't know; "." Missing)

© 2024 Yang and Toriola. *JAMA Health Forum.*

2017-2018	rhq031	{Have you/Has SP} had at least one menstrual period in the past 12 months? (Please do not include bleedings caused by medical conditions, hormone therapy, or surgeries.)<br>(1: yes, 2: No, 7: Refused; 9: Don't know, ".": Missing)
	rhd043	What is the reason that {you have/SP has} not had a period in the past 12 months?<br>(1: Pregnancy; 2: Breast feeding; 3: Hysterectomy; 7: Menopause/Change of life; 9: Other; 9: Other; 77: Refused; 99: Don't know; ".": Missing)
	rhq305	{Have you/Has SP} had both of {your/her} ovaries removed (either when {you/she} had {your/her} uterus removed or at another time)?<br>(1: Yes; 2: No; 7 Refused; 9: Don't know; "." Missing)
2017-2020	rhq031	{Have you/Has SP} had at least one menstrual period in the past 12 months? (Please do not include bleedings caused by medical conditions, hormone therapy, or surgeries.)<br>(1: yes, 2: No, 7: Refused; 9: Don't know, ".": Missing)
	rhd043	What is the reason that {you have/SP has} not had a period in the past 12 months?<br>(1: Pregnancy; 2: Breast feeding; 3: Hysterectomy; 7: Menopause/Change of life; 9: Other; 9: Other; 77: Refused; 99: Don't know; ".": Missing)
	rhq305	{Have you/Has SP} had both of {your/her} ovaries removed (either when {you/she} had {your/her} uterus removed or at another time)?<br>(1: Yes; 2: No; 7 Refused; 9: Don't know; "." Missing)

© 2024 Yang and Toriola. *JAMA Health Forum.*

eTable 2. Menopausal hormone therapy included in the analyses by Multum Lexicon Therapeutic Classification Scheme

Drug code	Drug name	Estrogen	Progesterone	Testosterone	1 Category ID	1 Category Name	2 Category ID	2 Category Name	3 Category ID	3 Category Name
a10899	ESTRADIOL; ESTRIOL; ESTRONE	Y	N	N	97	HORMONES/HORMONE MODIFIERS	101	SEX HORMONES	187	MISCELLANEOUS SEX HORMONES
a10900	ESTRADIOL; ESTRIOL	Y	N	N	97	HORMONES/HORMONE MODIFIERS	101	SEX HORMONES	187	MISCELLANEOUS SEX HORMONES
a11518	CONJUGATED ESTROGENS; PROGESTERONE	Y	Y	N	97	HORMONES/HORMONE MODIFIERS	101	SEX HORMONES	186	SEX HORMONE COMBINATIONS
a70248	ESTRIOL	Y	N	N	97	HORMONES/HORMONE MODIFIERS	101	SEX HORMONES	183	ESTROGENS
c00183	ESTROGENS - UNSPECIFIED	Y	N	N	97	HORMONES/HORMONE MODIFIERS	101	SEX HORMONES	183	ESTROGENS
d00284	MEDROXYPROGESTERONE	N	Y	N	20	ANTINEOPLASTICS	24	ANTINEOPLASTIC HORMONES		
					97	HORMONES/HORMONE MODIFIERS	101	SEX HORMONES	102	CONTRACEPTIVES
					97	HORMONES/HORMONE MODIFIERS	101	SEX HORMONES	185	PROGESTINS
d00534	ESTRONE	Y	N	N	97	HORMONES/HORMONE MODIFIERS	101	SEX HORMONES	183	ESTROGENS
d00537	ESTRADIOL	Y	N	N	97	HORMONES/HORMONE MODIFIERS	101	SEX HORMONES	183	ESTROGENS
d00541	CONJUGATED ESTROGENS	Y	N	N	97	HORMONES/HORMONE MODIFIERS	101	SEX HORMONES	183	ESTROGENS
d00542	ESTERIFIED ESTROGENS	Y	N	N	97	HORMONES/HORMONE MODIFIERS	101	SEX HORMONES	183	ESTROGENS

© 2024 Yang and Toriola. *JAMA Health Forum.*

d00543	ESTROPIPATE	Y	N	N	97	HORMONES/HORMONE MODIFIERS	101	SEX HORMONES	183	ESTROGENS
d00550	PROGESTERONE	N	Y	N	97	HORMONES/HORMONE MODIFIERS	101	SEX HORMONES	185	PROGESTINS
d03238	ETHINYL ESTRADIOL; NORETHINDRONE	Y	Y	N	97	HORMONES/HORMONE MODIFIERS	101	SEX HORMONES	102	CONTRACEPTIVES
					97	HORMONES/HORMONE MODIFIERS	101	SEX HORMONES	186	SEX HORMONE COMBINATIONS
d03245	ESTERIFIED ESTROGENS; METHYLTESTOSTERONE	Y	N	Y	97	HORMONES/HORMONE MODIFIERS	101	SEX HORMONES	186	SEX HORMONE COMBINATIONS
d03389	ESTRADIOL; TESTOSTERONE	Y	N	Y	97	HORMONES/HORMONE MODIFIERS	101	SEX HORMONES	186	SEX HORMONE COMBINATIONS
d03819	CONJUGATED ESTROGENS; MEDROXYPROGESTERONE	Y	Y	N	97	HORMONES/HORMONE MODIFIERS	101	SEX HORMONES	186	SEX HORMONE COMBINATIONS
d04210	ESTRADIOL TOPICAL	Y	N	N	97	HORMONES/HORMONE MODIFIERS	101	SEX HORMONES	183	ESTROGENS
					133	TOPICAL AGENTS	151	VAGINAL PREPARATIONS	269	MISCELLANEOUS VAGINAL AGENTS
d04213	PROGESTERONE TOPICAL	N	Y	N	97	HORMONES/HORMONE MODIFIERS	101	SEX HORMONES	185	PROGESTINS
d04375	ESTRADIOL; NORETHINDRONE	Y	Y	N	97	HORMONES/HORMONE MODIFIERS	101	SEX HORMONES	186	SEX HORMONE COMBINATIONS
d04396	CONJUGATED ESTROGENS TOPICAL	Y	N	N	97	HORMONES/HORMONE MODIFIERS	101	SEX HORMONES	183	ESTROGENS

© 2024 Yang and Toriola. *JAMA Health Forum.*

					133	TOPICAL AGENTS	151	VAGINAL PREPARATION S	269	MISCELLANEOUS VAGINAL AGENTS
d04506	ESTRADIOL; NORGESTIMATE	Y	Y	N	97	HORMONES/HOR MONE MODIFIERS	101	SEX HORMONES	186	SEX HORMONE COMBINATIONS
d04721	ESTRADIOL; MEDROXYPROGESTE RONE	Y	Y	N	97	HORMONES/HOR MONE MODIFIERS	101	SEX HORMONES	102	CONTRACEPTIVES
					97	HORMONES/HOR MONE MODIFIERS	101	SEX HORMONES	186	SEX HORMONE COMBINATIONS
d04772	ETONOGESTREL	N	Y	N	97	HORMONES/HOR MONE MODIFIERS	101	SEX HORMONES	102	CONTRACEPTIVES
					97	HORMONES/HOR MONE MODIFIERS	101	SEX HORMONES	185	PROGESTINS
d04773	ETHINYL ESTRADIOL; ETONOGESTREL	Y	Y	N	97	HORMONES/HOR MONE MODIFIERS	101	SEX HORMONES	102	CONTRACEPTIVES
d04779	ETHINYL ESTRADIOL; NORELGESTROMIN	Y	Y	N	97	HORMONES/HOR MONE MODIFIERS	101	SEX HORMONES	102	CONTRACEPTIVES
d04914	ESTRADIOL; LEVONORGESTREL	Y	Y	N	97	HORMONES/HOR MONE MODIFIERS	101	SEX HORMONES	186	SEX HORMONE COMBINATIONS
d05027	ESTRIOL	Y	N	N	97	HORMONES/HOR MONE MODIFIERS	101	SEX HORMONES	183	ESTROGENS
d05530	DROSPIRENONE; ESTRADIOL	N	Y	N	97	HORMONES/HOR MONE MODIFIERS	101	SEX HORMONES	186	SEX HORMONE COMBINATIONS

© 2024 Yang and Toriola. *JAMA Health Forum.*

eTable 3.1 Sociodemographic characteristics of postmenopausal women from the NHANES overall and by age group, 2017-March 2020 pre-pandemic[a]

	Study population, No.		Percentage			
				Age group		
	Sample	Weighted	Overall	<52	52-65	≥65
**Total**	1892	47868404		6.6%	46.2%	47.1%
**Race/ethnicity**						
Non-Hispanic white	791	34345146	71.7%	67.7%	66.8%	77.1%
Non-Hispanic black	466	4573780	9.6%	8.7%	11.3%	7.9%
Hispanic	370	4979460	10.4%	8.5%	12.3%	8.8%
Other[b]	265	3970018	8.3%	15.2%	9.5%	6.1%
**Family income to poverty ratio**						
<1.3	418	6890594	14.4%	16.0%	16.4%	12.2%
1.3 - <3.5	650	15857318	33.1%	23.9%	28.0%	39.4%
>=3.5	541	19403746	40.5%	51.4%	43.2%	36.4%
Missing	283	5716745	11.9%	8.7%	12.3%	12.0%
**Educational attainment**						
<High school	381	5121603	10.7%	9.8%	8.2%	13.3%
High school	486	14192149	29.6%	17.8%	31.2%	29.8%
>High school	1022	28515377	59.6%	72.4%	60.6%	56.7%
Missing	3	39274	0.1%	0.0%	0.0%	0.1%
**Health Insurance**						
No insurance	159	2754079	5.8%	7.9%	9.8%	1.5%
Any insurance	764	15691971	32.8%	24.2%	24.1%	42.5%
Private insurance	967	29399892	61.4%	67.9%	65.9%	56.1%
Missing	2	22461	0.0%	0.0%	0.1%	0.0%
**Marital status**						
Living alone	975	21031096	43.9%	44.1%	36.4%	51.3%
Living with someone	913	26791409	56.0%	55.9%	63.6%	48.5%
Missing	4	45899	0.1%	0.0%	0.0%	0.2%
**Body mass index**						
<25 kg/m^2	376	10631004	22.2%	26.3%	23.8%	20.1%
25-<30 kg/m^2	506	12841216	26.8%	24.0%	27.3%	26.8%
≥30 kg/m^2	791	19448517	40.6%	46.9%	41.0%	39.4%
Missing	219	4947666	10.3%	2.7%	8.0%	13.7%
**Smoke status**						
Never	1186	28633065	59.8%	53.0%	57.6%	62.9%
Past	464	12941214	27.0%	18.5%	25.3%	29.9%
Current	241	6284895	13.1%	28.5%	17.1%	7.1%
Missing	1	9230	0.0%	0.0%	0.0%	0.0%

© 2024 Yang and Toriola. *JAMA Health Forum.*

a All data are weighted to be US nationally representative.
b “Other” race/ethnicity includes race/ethnicity other than non-Hispanic white, non-Hispanic Black and Hispanic, including multiracial.

© 2024 Yang and Toriola. *JAMA Health Forum.*

eTable 3.2 Sociodemographic characteristics of postmenopausal women from the NHANES overall and by race/ethnicity, 2017-March 2020 pre-pandemic[a]

	Study population, No.		Percentage				
				Racial/ethnic group			
	Sample	Weighted	Overall	Non-Hispanic White	Non-Hispanic Black	Hispanic	Others[b]
**Total**	1892	47868404		71.7%	9.6%	10.4%	8.3%
**Age group**							
<52	110	3173688	6.6%	6.3%	6.0%	5.4%	12.1%
52-65	854	22130542	46.2%	43.1%	54.9%	54.8%	52.9%
≥65	928	22564173	47.1%	50.7%	39.1%	39.8%	34.9%
**Family income to poverty ratio**							
<1.3	418	6890594	14.4%	10.4%	29.2%	22.2%	21.6%
1.3 - <3.5	650	15857318	33.1%	33.6%	30.8%	35.2%	29.0%
≥3.5	541	19403746	40.5%	45.1%	24.0%	25.2%	39.0%
Missing	283	5716745	11.9%	10.8%	16.0%	17.3%	10.4%
**Educational attainment**							
<High school	381	5121603	10.7%	6.6%	15.9%	33.5%	11.8%
High school	486	14192149	29.6%	30.6%	32.1%	23.1%	26.7%
>High school	1022	28515377	59.6%	62.8%	51.8%	43.1%	61.5%
Missing	3	39274	0.1%	0.0%	0.2%	0.3%	0.0%
**Health insurance**							
No insurance	159	2754079	5.8%	4.1%	9.1%	13.8%	6.0%
Any insurance	764	15691971	32.8%	29.2%	42.7%	42.6%	40.2%
Private insurance	967	29399892	61.4%	66.7%	47.7%	43.6%	53.8%
Missing	2	22461	0.0%	0.0%	0.5%	0.0%	0.0%
**Marital status**							
Living alone	975	21031096	43.9%	41.3%	64.3%	47.8%	38.1%
Living with someone	913	26791409	56.0%	58.6%	35.1%	52.2%	61.9%
Missing	4	45899	0.1%	0.1%	0.6%	0.0%	0.0%
**Body mass index**							
<25 kg/m^2	376	10631004	22.2%	24.0%	12.1%	12.0%	31.6%
25-<30 kg/m^2	506	12841216	26.8%	26.5%	21.1%	35.2%	25.4%
≥30 kg/m^2	791	19448517	40.6%	39.1%	57.5%	43.6%	30.6%
Missing	219	4947666	10.3%	10.4%	9.3%	9.2%	12.5%
**Smoke status**							
Never	1186	28633065	59.8%	56.9%	58.5%	73.0%	69.6%
Past	464	12941214	27.0%	29.7%	23.4%	19.6%	17.5%
Current	241	6284895	13.1%	13.3%	18.0%	7.4%	12.9%
Missing	1	9230	0.0%	0.0%	0.2%	0.0%	0.0%

a All data are weighted to be US nationally representative.

© 2024 Yang and Toriola. *JAMA Health Forum.*

b “Other” race/ethnicity includes race/ethnicity other than non-Hispanic white, non-Hispanic Black and Hispanic, including multiracial.

eTable 4.1 Sample size of postmenopausal women from the NHANES by study cycle 1999-March 2020 pre-pandemic[a]

	Overall		By race/ethnicity							
			Non-Hispanic White		Non-Hispanic Black		Hispanic		Other[b]	
**Study cycle**	**Sample**	**Weighted**	**Sample**	**Weighted**	**Sample**	**Weighted**	**Sample**	**Weighted**	**Sample**	**Weighted**
**1999-2000**	1217	40476431	592	30481072	225	3708877	366	4566894	34	1719588
**2001-2002**	1356	43892505	827	34145021	225	4032246	261	3765271	43	1949966
**2003-2004**	1234	43122373	737	34070213	209	4368341	245	2621086	43	2062733
**2005-2006**	1008	43323129	588	34672794	229	4625527	162	2536319	29	1488489
**2007-2008**	1482	45899801	741	34720785	316	5117280	379	3801994	46	2259742
**2009-2010**	1373	45343642	725	34608704	235	4779115	366	3713768	47	2242055
**2011-2012**	1277	50998476	512	38186997	374	5613120	241	4364274	150	2834085
**2013-2014**	1112	42329550	556	32089983	190	3744442	230	3865020	136	2630104
**2015-2016**	1097	46325578	417	34603624	191	4039336	372	4435585	117	3247033
**2017-2020**	1892	47868404	791	34345146	466	4573780	370	4979460	265	3970018

a All data are weighted to be US nationally representative.

b “Other” race/ethnicity includes race/ethnicity other than non-Hispanic white, non-Hispanic Black and Hispanic, including multiracial.

© 2024 Yang and Toriola. *JAMA Health Forum.*

eTable 4.2 Sample size of postmenopausal women from the NHANES with complete data on correlates by study cycle, 1999-March 2020 pre-pandemic[a]

	Overall		By race/ethnicity							
			Non-Hispanic White		Non-Hispanic Black		Hispanic		Other[b]	
**Study cycle**	**Sample**	**Weighted**	**Sample**	**Weighted**	**Sample**	**Weighted**	**Sample**	**Weighted**	**Sample**	**Weighted**
**1999-2000**	788	27737240	376	20980088	148	2417282	241	3242824	23	1097046
**2001-2002**	1015	35046969	605	27365467	174	3283653	206	3020043	30	1377805
**2003-2004**	1037	36835932	606	28952024	182	3820657	214	2349946	35	1713305
**2005-2006**	891	39315655	517	31548795	206	4212673	145	2323186	23	1231001
**2007-2008**	1233	39445698	629	30090389	265	4330665	303	3168133	36	1856510
**2009-2010**	1186	40909073	662	31956116	201	4100452	282	2901065	41	1951440
**2011-2012**	1072	44386853	444	33721687	309	4660032	197	3596200	122	2408933
**2013-2014**	975	38111835	502	29289711	168	3339737	193	3332268	112	2150119
**2015-2016**	900	40206993	358	30723042	155	3347862	299	3609945	88	2526144
**2017-2020**	1453	38587007	622	28116880	351	3503379	273	3789122	207	3177626

a All data are weighted to be US nationally representative.

b "Other" race/ethnicity includes race/ethnicity other than non-Hispanic white, non-Hispanic Black and Hispanic, including multiracial.

© 2024 Yang and Toriola. *JAMA Health Forum.*

eTable 5. Menopausal hormone therapy formulations prescribed among US postmenopausal women ≥52 years 1999-March 2020 pre-pandemic[a]

Study cycle	Weighted prevalence, % (95% CI)									
	Estrogen only		Progestogen only		Estrogen + progestogen		Estrogen + testosterone		Estrogen + Progestogen + testosterone	
**1999-2000**	60.3	(49.5 to 70.1)	1.7	(0.4 to 6.1)	34.3	(25.1 to 44.8)	2.0	(1.1 to 3.5)	1.8	(0.5 to 6.9)
**2001-2002**	63.9	(56.3 to 70.9)	1.3	(0.3 to 4.8)	32.3	(26.3 to 39.0)	2.2	(0.7 to 6.5)	0.3	(0.0 to 2.2)
**2003-2004**	72.0	(63.5 to 79.2)	0.0		20.5	(12.3 to 32.3)	7.5	(3.7 to 14.6)	0.0	
**2005-2006**	68.7	(54.3 to 80.2)	0.0		26.0	(16.1 to 39.1)	5.3	(1.5 to 17.3)	0.0	
**2007-2008**	73.3	(62.6 to 91.9)	0.8	(0.1 to 5.2)	21.2	(12.8 to 32.9)	4.7	(1.5 to 13.9)	0.0	
**2009-2010**	61.9	(46.7 to 75.1)	4.6	(1.2 to 16.2)	32.2	(19.9 to 47.7)	1.3	(0.2 to 9.8)	0.0	
**2011-2012**	67.6	(51.0 to 80.7)			32.4	(19.3 to 49.0)	0.0		0.0	
**2013-2014**	50.3	(35.4 to 65.1)	7.3	(3.8 to 13.6)	24.4	(12.4 to 42.3)	13.5	(4.3 to 35.1)	4.5	(0.7 to 24.9)
**2015-2016**	41.8	(20.9 to 66.2)	22.5	(4.2 to 66.0)	35.7	(13.5 to 66.4)	0.0		0.0	
**2017-2020**	47.4	(30.1 to 65.4)	12.1	(3.9 to 32.1)	39.8	(20.1 to 63.5)	0.7	(0.1 to 4.9)	0.0	

a All data are weighted to be US nationally representative.

© 2024 Yang and Toriola. *JAMA Health Forum.*

eTable 6.1 Prevalence and trends in menopausal hormone therapy use among US postmenopausal women overall and by age group, 2009-March 2020 pre-pandemic[a]

Study cycle	Weighted prevalence, % (95% CI)							
	Overall		<52 yr		52-<65 yr		≥65 yr	
**2009-2010**	6.7	(5.0 to 8.9)	8.9	(4.7 to 16.1)	8.3	(5.4 to 12.5)	4.3	(2.5 to 7.3)
**2011-2012**	7.8	(6.0 to 10.1)	13.0	(8.6 to 19.1)	9.9	(6.4 to 15.1)	3.8	(1.9 to 7.2)
**2013-2014**	8.4	(6.4 to 10.9)	18.3	(12.2 to 26.5)	9.7	(6.7 to 13.8)	5.0	(3.4 to 7.3)
**2015-2016**	6.8	(4.1 to 11.2)	12.7	(5.9 to 25.5)	7.9	(4.1 to 14.5)	4.6	(2.6 to 8.0)
**2017-2020**	4.7	(3.4 to 6.5)	9.4	(3.9 to 21.1)	4.5	(2.9 to 6.7)	4.3	(2.7 to 6.7)
**P for trend**	0.015		0.539		0.004		0.945	
**2017-2020 vs. 2009-2010 Difference (95%CI)[b]**								
	-2.0	(-4.5 to 0.5)	0.52	(-9.46 to 10.5)	-3.83	(-9.76 to 0.20)	0.03	(-3.03 to 3.09)
**2017-2020 vs. 2009-2010 Prevalence Ratio (95%CI)[c]**								
	0.70	(0.45 to 1.09)	1.06	(0.36 to 3.11)	0.54	(0.29 to 0.98)	1.01	(0.49 to 2.06)

a All data are weighted to be US nationally representative.

b indicates the absolute change in prevalence of menopausal hormone therapy use between 2009-2010 and 2017-March 2020 pre-pandemic

c Indicate the relative change in prevalence of menopausal hormone therapy use between 2009-2010 and 2017-March 2020 pre-pandemic

© 2024 Yang and Toriola. *JAMA Health Forum.*

eTable 6.2 Prevalence and trends in menopausal hormone therapy use among US postmenopausal women by race/ethnicity, 2009-March 2020 pre-pandemic[a]

Study cycle	Weighted prevalence, % (95% CI)			
	Non-Hispanic White	Non-Hispanic Black	Hispanic	Others
**2009-2010**	7.9 (5.6 to 11.0)	2.9 (1.3 to 6.1)	2.0 (0.9 to 4.4)	4.5 (0.9 to 18.9)
**2011-2012**	9.5 (7.1 to 12.5)	2.7 (1.4 to 5.0)	4.5 (2.1 to 9.3)	0.6 (0.1 to 3.8)
**2013-2014**	9.5 (7.1 to 12.7)	5.1 (2.5 to 10.3)	4.4 (2.0 to 9.4)	5.5 (2.7 to 10.9)
**2015-2016**	8.0 (5.0 to 12.4)	1.5 (0.5 to 4.5)	2.0 (0.9 to 4.7)	7.9 (1.5 to 32.4)
**2017-2020**	5.8 (4.1 to 8.2)	0.5 (0.2 to 1.1)	2.6 (1.5 to 4.6)	2.8 (1.1 to 7.1)
**P for trend**	0.039	<0.001	0.542	0.450
**2017-2020 vs. 2009-2010 Difference (95%CI)[b]**				
	-2.0 (-5.5 to 1.3)	-2.4 (-4.6 to -0.1)	0.6 (-1.7 to 2.8)	-1.7 (-9.2 to 5.9)
**2017-2020 vs. 2009-2010 Prevalence Ratio (95%CI)[c]**				
	0.73 (0.45 to 1.21)	0.18 (0.06 to 0.54)	1.29 (0.48 to 3.51)	0.63 (0.10 to 3.96)

a All data are weighted to be US nationally representative.

b indicates the absolute change in prevalence of menopausal hormone therapy use between 2009-2010 and 2017-March 2020 pre-pandemic

c Indicate the relative change in prevalence of menopausal hormone therapy use between 2009-2010 and 2017-March 2020 pre-pandemic

© 2024 Yang and Toriola. *JAMA Health Forum.*

eTable 7. Sociodemographic characteristics of postmenopausal women from the NHANES, 2017-2018[a]

	Study population, No.		Percentage				
				Racial/ethnic group			
	Sample	Weighted	Overall	Non-Hispanic White	Non-Hispanic Black	Hispanic	Others[b]
**Total**	1148	46927126		71.6%	9.4%	10.7%	8.4%
**Age group**							
<52	75	3987071	8.5%	8.7%	6.9%	5.7%	12.2%
52-65	526	21998359	46.9%	43.2%	55.9%	57.1%	55.2%
≥65	547	20941696	44.6%	48.1%	37.2%	37.2%	32.6%
**Family income to poverty ratio**							
<1.3	252	6735141	14.4%	10.7%	26.7%	21.1%	23.5%
1.3 - <3.5	422	15893582	33.1%	35.0%	31.7%	34.3%	26.2%
≥3.5	317	18992777	40.5%	44.5%	24.8%	26.8%	41.1%
Missing	157	5305627	11.9%	9.9%	16.8%	17.7%	9.2%
**Educational attainment**							
<High school	234	4531287	10.7%	5.5%	16.0%	31.6%	10.3%
High school	303	14582495	29.6%	32.2%	32.2%	23.2%	30.1%
>High school	609	27778961	59.6%	62.3%	51.4%	44.9%	59.7%
Missing	2	34384	0.1%	0.0%	0.3%	0.4%	0.0%
**Health insurance**							
No insurance	104	3106972	5.8%	5.6%	7.7%	13.6%	5.3%
Any insurance	452	14892621	32.8%	27.7%	43.0%	42.6%	40.0%
Private insurance	590	28904700	61.4%	66.7%	48.8%	43.9%	54.8%
Missing	2	22833	0.0%	0.0%	0.5%	0.0%	0.0%
**Marital status**							
Living alone	564	19884314	43.9%	40.2%	63.4%	45.6%	33.0%
Living with someone	583	27028002	56.0%	59.8%	36.3%	54.4%	67.0%
Missing	1	14810	0.1%	0.0%	0.3%	0.0%	0.0%
**Body mass index**							
<25 kg/m^2	244	11568288	22.2%	27.2%	10.5%	14.2%	31.6%
25-<30 kg/m^2	327	12929517	26.8%	26.5%	24.8%	36.5%	28.6%
≥30 kg/m^2	484	18881359	40.6%	38.6%	59.0%	42.2%	30.4%
Missing	93	3547962	10.3%	7.7%	5.6%	7.1%	9.4%
**Smoke status**							
Never	743	30048766	59.8%	62.6%	59.7%	70.6%	72.8%
Past	262	11238242	27.0%	25.3%	22.4%	22.4%	16.1%
Current	143	5640119	13.1%	12.1%	17.9%	7.0%	11.0%

a All data are weighted to be US nationally representative.

b “Other” race/ethnicity includes race/ethnicity other than non-Hispanic white, non-Hispanic Black and Hispanic, including multiracial.

© 2024 Yang and Toriola. *JAMA Health Forum.*

eTable 8.1 Menopausal hormone therapy formulations prescribed among US postmenopausal women, 1999-2018[a]

Study cycle	Weighted prevalence, % (95% CI)				
	Estrogen only	Progestogen only	Estrogen + progestogen	Estrogen + testosterone	Estrogen + progestogen + testosterone
**1999-2000**	58.3 (49.8 to 66.3)	3.3 (1.0 to 10.2)	28.6 (22.2 to 36.1)	7.6 (3.9 to 14.3)	2.2 (0.7 to 6.2)
**2001-2002**	62.0 (54.6 to 68.8)	1.4 (0.5 to 4.2)	32.3 (25.1 to 40.3)	3.6 (2.0 to 6.5)	0.7 (0.2 to 3.0)
**2003-2004**	73.4 (65.2 to 80.3)	0.0	20.8 (14.9 to 28.4)	5.7 (2.9 to 11.1)	0.0
**2005-2006**	74.2 (59.3 to 85.0)	0.0	18.3 (10.5 to 29.9)	5.4 (1.7 to 15.8)	2.1 (0.3 to 12.9)
**2007-2008**	78.0 (69.2 to 84.9)	0.5 (0.1 to 3.8)	18.2 (11.0 to 28.5)	3.3 (1.1 to 9.6)	0.0
**2009-2010**	57.9 (46.1 to 69.0)	3.6 (0.9 to 13.5)	33.7 (22.7 to 46.8)	4.7 (0.9 to 21.7)	0.0
**2011-2012**	65.5 (48.2 to 79.5)	4.0 (0.8 to 18.2)	28.6 (15.3 to 46.9)	1.9 (0.2 to 12.7)	0.0
**2013-2014**	55.2 (43.4 to 66.5)	8.1 (4.4 to 14.6)	22.0 (11.7 to 37.6)	11.1 (4.0 to 27.3)	3.5 (0.5 to 20.5)
**2015-2016**	38.2 (17.3 to 64.6)	25.0 (4.7 to 69.2)	36.8 (13.2 to 69.0)	0.0	0.0
**2017-2018**	57.3 (40.9 to 72.2)	11.9 (2.7 to 39.4)	30.1 (13.0 to 55.3)	0.7 (0.1 to 4.8)	0.0

a All data are weighted to be US nationally representative.

© 2024 Yang and Toriola. *JAMA Health Forum.*

eTable 8.2 Menopausal hormone therapy formulations prescribed among US postmenopausal women with no history of breast cancer, 1999-March 2020 pre-pandemic[a]

Study cycle	Weighted prevalence, % (95% CI)				
	Estrogen only	Progestogen only	Estrogen + progestogen	Estrogen + testosterone	Estrogen + progestogen+ testosterone
**1999-2000**	58.3 (49.8 to 66.3)	3.3 (1.0 to 10.2)	28.6 (22.2 to 36.1)	7.6 (3.9 to 14.3)	2.2 (0.7 to 6.2)
**2001-2002**	61.9 (54.5 to 68.8)	1.5 (0.5 to 4.3)	32.7 (25.2 to 41.2)	3.2 (1.5 to 6.8)	0.7 (0.2 to 3.0)
**2003-2004**	73.2 (65.3 to 79.8)	0.0	21.0 (15.2 to 28.4)	5.8 (2.9 to 11.2)	0.0
**2005-2006**	74.2 (59.3 to 85.0)	0.0	18.3 (10.5 to 29.9)	5.4 (1.7 to 15.7)	2.1 (0.3 to 12.9)
**2007-2008**	78.0 (69.2 to 84.9)	0.5 (0.1 to 3.8)	18.2 (11.0 to 28.5)	3.3 (1.1 to 9.6)	0.0
**2009-2010**	57.9 (46.1 to 69.0)	3.6 (0.9 to 13.5)	33.7 (22.8 to 46.8)	4.7 (0.9 to 21.7)	0.0
**2011-2012**	65.5 (48.2 to 79.5)	4.0 (0.8 to 18.2)	28.6 (15.4 to 46.9)	1.9 (0.2 to 12.7)	0.0
**2013-2014**	55.2 (43.4 to 66.5)	8.1 (4.4 to 14.6)	22.0 (11.7 to 37.6)	11.1 (4.0 to 27.3)	3.5 (0.5 to 20.5)
**2015-2016**	38.2 (17.3 to 64.5)	25.0 (4.7 to 69.1)	36.8 (13.2 to 69.0)	0.0	0.0
**2017-2020**	56.2 (38.9 to 72.1)	11.2 (4.0 to 27.5)	32.0 (16.9 to 52.2)	0.7 (0.1 to 4.5)	0.0

a All data are weighted to be US nationally representative.

© 2024 Yang and Toriola. *JAMA Health Forum.*

eTable 8.3 Menopausal hormone therapy formulations prescribed among US postmenopausal women with complete data on corelates, 1999-March 2020 pre-pandemic[a]

Study cycle	Weighted prevalence, % (95% CI)				
	Estrogen only	Progestogen only	Estrogen + progestogen	Estrogen + testosterone	Estrogen + progestogen + testosterone
**1999-2000**	58.1 (50.1 to 65.7)	3.5 (1.1 to 10.5)	27.3 (20.7 to 35.0)	9.6 (5.1 to 17.4)	1.6 (0.4 to 5.7)
**2001-2002**	62.1 (54.3 to 69.4)	1.3 (0.4 to 4.4)	32.2 (24.2 to 41.4)	3.7 (2.2 to 6.1)	0.8 (0.2 to 3.4)
**2003-2004**	73.3 (65.3 to 80.0)	0.0	20.9 (14.9 to 28.5)	5.8 (2.8 to 11.7)	0.0
**2005-2006**	74.5 (59.2 to 85.5)	0.0	17.8 (10.0 to 29.8)	5.5 (1.8 to 16.0)	2.2 (0.3 to 13.1)
**2007-2008**	79.3 (70.4 to 86.0)	0.6 (0.1 to 4.2)	16.9 (10.1 to 27.0)	3.2 (1.0 to 10.1)	0.0
**2009-2010**	60.0 (47.5 to 71.4)	3.8 (1.0 to 14.1)	31.3 (20.0 to 45.3)	4.9 (0.9 to 22.7)	0.0
**2011-2012**	63.6 (44.9 to 78.9)	4.3 (0.8 to 19.0)	30.2 (16.0 to 49.5)	2.0 (0.2 to 14.0)	0.0
**2013-2014**	54.2 (43.0 to 65.0)	8.8 (4.8 to 15.5)	21.3 (11.1 to 37.0)	12.0 (4.2 to 29.5)	3.8 (0.6 to 21.7)
**2015-2016**	37.2 (16.7 to 63.8)	26.9 (5.1 to 71.5)	35.8 (12.0 to 69.6)	0.0	0.0
**2017-2020**	51.0 (33.9 to 67.9)	9.6 (2.7 to 28.6)	38.7 (20.7 to 60.5)	0.7 (0.1 to 4.6)	0.0

a All data are weighted to be US nationally representative.

© 2024 Yang and Toriola. *JAMA Health Forum.*

eTable 9.1 Prevalence and trends in menopausal hormone therapy use among US postmenopausal women overall and by age group, 1999-2018[a]

	Weighted prevalence, % (95% CI)											
**Study cycle**	**Overall**			**<52 yr**			**52-<65 yr**			**≥65 yr**		
**1999-2000**	26.9	(22.6 to	31.7)	32.9	(25.0 to	42.0)	35.9	(28.7 to	43.9)	14.9	(11.5 to	18.9)
**2001-2002**	29.5	(25.2 to	34.2)	26.2	(18.7 to	35.5)	38.5	(31.9 to	45.5)	23.2	(18.7 to	28.4)
**2003-2004**	16.2	(13.9 to	18.9)	20.0	(14.1 to	27.5)	21.8	(18.3 to	25.8)	9.5	(6.5 to	13.6)
**2005-2006**	12.0	(10.1 to	14.2)	26.8	(17.6 to	38.5)	14.8	(10.7 to	20.1)	3.5	(2.2 to	5.5)
**2007-2008**	9.8	(7.6 to	12.5)	17.9	(12.6 to	24.7)	8.7	(6.0 to	12.4)	7.6	(4.9 to	11.5)
**2009-2010**	6.7	(5.0 to	8.9)	8.9	(4.7 to	16.1)	8.3	(5.4 to	12.5)	4.3	(2.5 to	7.3)
**2011-2012**	7.8	(6.0 to	10.1)	13.0	(8.6 to	19.1)	9.9	(6.4 to	15.1)	3.8	(1.9 to	7.2)
**2013-2014**	8.4	(6.4 to	10.9)	18.3	(12.2 to	26.5)	9.7	(6.7 to	13.8)	5.0	(3.4 to	7.3)
**2015-2016**	6.8	(4.1 to	11.2)	12.7	(5.9 to	25.5)	7.9	(4.1 to	14.5)	4.6	(2.6 to	8.0)
**2017-2018**	5.9	(4.1 to	8.7)	15.1	(4.2 to	42.1)	4.7	(2.9 to	7.6)	5.5	(3.5 to	8.6)
**P for trend**	<.001			<.001			<.001			<.001		
**2017-2018vs. 1999-2000 Difference (95%CI)[b]**												
	-21.0	(-26. to	-15.7)	-17.8	(-38.4 to	2.9)	-31.2	(-39.5 to	-22.9)	-9.4	(-14.0 to	-4.8)
**2017-2018 vs. 1999-2000 Prevalence Ratio (95%CI)[c]**												
	0.22	(0.14 to	0.34)	0.46	(0.13 to	1.62)	0.13	(0.08 to	0.23)	0.37	(0.22 to	0.63)

a All data are weighted to be US nationally representative.

b indicates the absolute change in prevalence of MHT use between 1999-2000 and 2017-2018.

c indicates the relative change in prevalence of MHT use between 1999-2000 and 2017-2018.

© 2024 Yang and Toriola. *JAMA Health Forum.*

eTable 9.2 Prevalence and trends in menopausal hormone therapy use among US postmenopausal women by race/ethnicity, 1999-2018[a]

	Weighted prevalence, % (95% CI)											
Study cycle	Overall			Non-Hispanic White			Non-Hispanic Black			Hispanic		
**1999-2000**	31.4	(27.1 to	36.1)	11.9	(8.5 to	16.3)	13.8	(8.5 to	21.7)	13.5	(3.3 to	41.5)
**2001-2002**	32.7	(28.3 to	37.3)	17.8	(11.8 to	26.0)	14.8	(9.9 to	21.5)	26.8	(12.7 to	48.2)
**2003-2004**	17.5	(14.8 to	20.5)	12.7	(8.4 to	18.8)	8.7	(4.4 to	16.4)	12.8	(4.8 to	30.2)
**2005-2006**	13.4	(11.1 to	16.2)	7.3	(3.0 to	16.9)	4.4	(1.7 to	10.8)	6.1	(1.2 to	25.3)
**2007-2008**	11.2	(8.6 to	14.5)	3.2	(1.6 to	6.3)	6.0	(3.9 to	9.0)	9.0	(2.2 to	30.8)
**2009-2010**	7.9	(5.6 to	11.0)	2.9	(1.3 to	6.1)	2.0	(0.9 to	4.4)	4.5	(0.9 to	18.9)
**2011-2012**	9.5	(7.1 to	12.5)	2.7	(1.4 to	5.0)	4.5	(2.1 to	9.3)	0.6	(0.1 to	3.8)
**2013-2014**	9.5	(7.1 to	12.7)	5.1	(2.5 to	10.3)	4.4	(2.0 to	9.4)	5.5	(2.7 to	10.9)
**2015-2016**	8.0	(5.0 to	12.4)	1.5	(0.5 to	4.5)	2.0	(0.9 to	4.7)	7.9	(1.5 to	32.4)
**2017-2018**	7.4	(4.8 to	11.2)	1.3	(0.6 to	2.8)	3.0	(1.4 to	6.2)	2.8	(0.9 to	8.2)
**P for trend**	<.001			<.001			<.001			0.018		
**2017-2018vs. 1999-2000 Difference (95%CI)[b]**												
	-24.1	(-29.7 to	-18.4)	-10.6	(-14.7 to	-6.4)	-10.7	(-17.9 to	-3.7)	-10.7	(-29.3 to	8.0)
**2017-2018 vs. 1999-2000 Prevalence Ratio (95%CI)[c]**												
	0.23	(0.15 to	0.37)	0.11	(0.04 to	0.26)	0.22	(0.09 to	0.54)	0.21	(0.04 to	1.22)

a All data are weighted to be US nationally representative.
b indicates the absolute change in prevalence of MHT use between 1999-2000 and 2017-2018.
c indicates the relative change in prevalence of MHT use between 1999-2000 and 2017-2018.

© 2024 Yang and Toriola. *JAMA Health Forum.*

eTable 9.3 Prevalence and trends in menopausal hormone therapy use among US postmenopausal women with no history of breast cancer overall and by age group, 1999-March 2020 pre-pandemic[a]

Study cycle	Weighted prevalence (95% CI)											
	**Overall**			**<52 yr**			**52-<65 yr**			**≥65 yr**		
**1999-2000**	27.9	(23.7 to	32.6)	33.2	(25.2 to	42.3)	36.6	(29.3 to	44.5)	16.1	(12.6 to	20.2)
**2001-2002**	30.5	(26.1 to	35.4)	26.7	(18.9 to	36.1)	39.3	(32.6 to	46.5)	24.7	(20.0 to	30.0)
**2003-2004**	16.9	(14.4 to	19.7)	20.5	(14.4 to	28.3)	22.0	(18.4 to	26.0)	10.3	(7.1 to	14.7)
**2005-2006**	12.8	(10.7 to	15.1)	27.7	(17.9 to	40.2)	15.5	(11.3 to	21.0)	3.8	(2.4 to	6.0)
**2007-2008**	10.3	(8.0 to	13.2)	18.6	(13.1 to	25.7)	9.1	(6.3 to	12.9)	8.2	(5.3 to	12.4)
**2009-2010**	7.1	(5.4 to	9.5)	9.0	(4.8 to	16.3)	8.6	(5.6 to	13.0)	4.8	(2.7 to	8.3)
**2011-2012**	8.1	(6.3 to	10.5)	13.0	(8.7 to	19.1)	10.1	(6.6 to	15.2)	4.0	(2.1 to	7.7)
**2013-2014**	9.0	(6.9 to	11.7)	18.3	(12.2 to	26.5)	10.2	(7.0 to	14.6)	5.5	(3.7 to	8.0)
**2015-2016**	7.3	(4.3 to	12.0)	13.0	(6.0 to	25.9)	8.3	(4.3 to	15.4)	5.0	(2.9 to	8.6)
**2017-2018**	4.7	(3.3 to	6.8)	10.1	(4.2 to	22.5)	4.0	(2.3 to	6.8)	4.8	(3.0 to	7.4)
**P for trend**	<.001			0.062			<0.001			0.002		
**2017-2020 vs. 1999-2000 Difference (95%CI)b**												
	-23.2	(-28.1 to	-18.3)	-23.1	(-35.6 to	-10.6)	-32.6	(-40.7 to	-24.5)	-11.3	(-15.7 to	-6.9)
**2017-2020 vs. 1999-2000 Prevalence Ratio (95%CI)[c]**												
	0.19	(0.11 to	0.26)	0.30	(0.12 to	0.76)	0.11	(0.06 to	0.20)	0.30	(0.18 to	0.50)

a All data are weighted to be US nationally representative.
b indicates the absolute change in prevalence of MHT use between 1999-2000 and 2017-March 2020 pre-pandemic
c indicates the relative change in prevalence of MHT use between 1999-2000 and 2017-March 2020 pre-pandemic

© 2024 Yang and Toriola. *JAMA Health Forum.*

eTable 9.4 Prevalence and trends in menopausal hormone therapy use among US postmenopausal women with no history of breast cancer by race/ethnicity, 1999-March 2020 pre-pandemic[a]

Study cycle	Weighted prevalence (95% CI) Non-Hispanic White			Non-Hispanic Black			Hispanic			Others		
**1999-2000**	32.8	(28.7 to	37.1)	12.3	(8.7 to	17.0)	14.0	(8.6 to	22.0)	13.9	(3.5 to	42.0)
**2001-2002**	34.2	(29.6 to	39.1)	17.6	(11.7 to	25.5)	14.9	(9.9 to	21.6)	26.8	(12.7 to	48.2)
**2003-2004**	18.2	(15.4 to	21.4)	13.2	(8.6 to	19.7)	8.8	(4.4 to	16.6)	13.4	(5.0 to	31.1)
**2005-2006**	14.4	(11.9 to	17.3)	7.7	(3.1 to	17.6)	4.5	(1.8 to	10.9)	6.1	(1.2 to	25.3)
**2007-2008**	11.9	(9.1 to	15.4)	3.3	(1.6 to	6.5)	6.2	(4.0 to	9.4)	9.7	(2.3 to	32.6)
**2009-2010**	8.5	(6.0 to	11.9)	3.0	(1.4 to	6.3)	2.1	(0.9 to	4.5)	4.5	(0.9 to	19.0)
**2011-2012**	9.9	(7.4 to	13.0)	2.8	(1.5 to	5.2)	4.8	(2.2 to	9.9)	0.6	(0.1 to	4.0)
**2013-2014**	10.2	(7.6 to	13.6)	5.4	(2.6 to	11.0)	4.6	(2.1 to	9.7)	5.9	(2.9 to	11.7)
**2015-2016**	8.6	(5.3 to	13.5)	1.6	(0.5 to	4.7)	2.1	(0.9 to	4.9)	8.1	(1.5 to	32.9)
**2017-2018**	5.8	(3.9 to	8.7)	0.5	(0.2 to	1.2)	2.7	(1.5 to	4.8)	3.0	(1.2 to	7.4)
**P for trend**		<.001			0.001			0.005			0.037	
**2017-2020 vs. 1999-2000 Difference (95%CI)b**												
	-26.9	(-31.9 to	-22.0)	-11.8	(-16.0 to	-7.5)	-11.3	(-18.3 to	-4.3)	-10.9	(-29.6 to	7.7)
**2017-2020 vs. 1999-2000 Prevalence Ratio (95%CI)[c]**												
	0.18	(0.12 to	0.27)	0.04	(0.02 to	0.10)	0.19	(0.09 to	0.42)	0.21	(0.04 to	1.10)

a All data are weighted to be US nationally representative.
b indicates the absolute change in prevalence of MHT use between 1999-2000 and 2017-March 2020 pre-pandemic
c indicates the relative change in prevalence of MHT use between 1999-2000 and 2017-March 2020 pre-pandemic

© 2024 Yang and Toriola. *JAMA Health Forum.*

eTable 9.5 Prevalence and trends in menopausal hormone therapy use among US postmenopausal women with complete data on correlates overall and by age group, 1999-March 2020 pre-pandemic[a]

	Weighted prevalence (95% CI)											
**Study cycle**	**Overall**			**<52 yr**			**52-<65 yr**			**≥65 yr**		
**1999-2000**	31.1	(26.4 to	36.1)	34.6	(25.8 to	44.6)	39.4	(31.5 to	47.9)	18.8	(14.0 to	24.8)
**2001-2002**	32.1	(27.1 to	37.5)	26.5	(18.3 to	36.6)	41.1	(34.4 to	48.1)	26.3	(20.6 to	32.9)
**2003-2004**	17.6	(15.3 to	20.1)	21.5	(15.5 to	29.0)	23.4	(19.6 to	27.6)	10.0	(7.2 to	13.6)
**2005-2006**	13.0	(10.8 to	15.5)	27.8	(18.2 to	40.1)	15.7	(11.3 to	21.3)	3.5	(2.0 to	6.0)
**2007-2008**	10.4	(8.1 to	13.3)	17.3	(11.9 to	24.5)	9.6	(6.4 to	14.2)	8.0	(5.1 to	12.2)
**2009-2010**	7.1	(5.4 to	9.3)	8.4	(4.4 to	15.3)	9.0	(5.9 to	13.5)	4.5	(2.6 to	7.6)
**2011-2012**	8.5	(6.6 to	11.0)	13.6	(8.3 to	21.5)	10.2	(6.5 to	15.6)	4.6	(2.3 to	8.9)
**2013-2014**	8.7	(6.5 to	11.5)	19.1	(12.9 to	27.2)	10.0	(6.8 to	14.5)	4.9	(3.1 to	7.5)
**2015-2016**	7.2	(4.2 to	12.3)	12.9	(5.9 to	25.8)	7.9	(4.0 to	15.0)	5.0	(2.7 to	9.2)
**2017-2018**	5.4	(3.8 to	7.8)	9.9	(3.9 to	23.0)	5.1	(3.2 to	8.0)	5.1	(3.1 to	8.1)
**P for trend**	<.001			0.074			<0.001			0.014		
**2017-2020 vs. 1999-2000 Difference (95%CI)b**												
	-25.6	(-31.0 to	-20.2)	-24.7	(-38.1 to	-11.4)	-34.3	(-43.1 to	-25.5)	-13.7	(-19.8 to	-7.7)
**2017-2020 vs. 1999-2000 Prevalence Ratio (95%CI)[c]**												
	0.18	(0.12 to	0.26)	0.29	(0.11 to	0.75)	0.13	(0.08 to	0.22)	0.27	(0.15 to	0.48)

a All data are weighted to be US nationally representative.

b indicates the absolute change in prevalence of MHT use between 1999-2000 and 2017-March 2020 pre-pandemic

c indicates the relative change in prevalence of MHT use between 1999-2000 and 2017-March 2020 pre-pandemic

© 2024 Yang and Toriola. *JAMA Health Forum.*

eTable 9.6 Prevalence and trends in menopausal hormone therapy use among US postmenopausal women with complete data on correlates by race/ethnicity, 1999-March 2020 pre-pandemic[a]

	Weighted prevalence (95% CI)											
**Study cycle**	**Non-Hispanic White**			**Non-Hispanic Black**			**Hispanic**			**Others**		
**1999-2000**	37.3	(32.5 to	42.4)	11.4	(7.6 to	16.7)	12.9	(7.5 to	21.3)	8.5	(1.7 to	33.5)
**2001-2002**	34.9	(29.7 to	40.6)	20.5	(14.0 to	29.0)	17.1	(11.3 to	24.9)	35.2	(15.6 to	61.4)
**2003-2004**	19.0	(16.5 to	21.7)	13.0	(7.5 to	21.5)	9.3	(4.6 to	17.9)	15.4	(6.0 to	34.2)
**2005-2006**	14.5	(11.8 to	17.6)	8.0	(3.3 to	18.4)	4.8	(1.8 to	11.8)	7.3	(1.5 to	29.4)
**2007-2008**	11.9	(9.1 to	15.4)	3.2	(1.3 to	7.5)	6.7	(4.2 to	10.7)	9.1	(1.7 to	36.1)
**2009-2010**	8.4	(6.0 to	11.7)	3.3	(1.6 to	6.9)	1.8	(0.7 to	4.6)	1.2	(0.2 to	7.0)
**2011-2012**	10.2	(7.8 to	13.2)	3.3	(1.8 to	6.0)	5.2	(2.4 to	10.7)	0.7	(0.1 to	4.5)
**2013-2014**	9.6	(7.0 to	13.1)	5.8	(2.8 to	11.4)	5.1	(2.4 to	10.8)	5.8	(2.8 to	11.6)
**2015-2016**	8.3	(5.0 to	13.3)	1.8	(0.6 to	5.5)	1.5	(0.4 to	5.6)	10.1	(2.0 to	37.9)
**2017-2018**	6.7	(4.6 to	9.7)	0.6	(0.2 to	1.7)	3.1	(1.7 to	5.6)	2.5	(0.8 to	7.0)
**P for trend**	<.001			0.001			0.014			0.040		
**2017-2020 vs. 1999-2000 Difference (95%CI)b**												
	-30.6	(-36.3 to	-24.9)	-10.8	(-15.5 to	-6.2)	-9.8	(-17.0 to	-2.6)	-6.0	(-19.8 to	7.7)
**2017-2020 vs. 1999-2000 Prevalence Ratio (95%CI)[c]**												
	0.18	(0.12 to	0.27)	0.05	(0.02 to	0.16)	0.24	(0.11 to	0.55)	0.29	(0.04 to	2.00)

a All data are weighted to be US nationally representative.
b indicates the absolute change in prevalence of MHT use between 1999-2000 and 2017-March 2020 pre-pandemic
c indicates the relative change in prevalence of MHT use between 1999-2000 and 2017-March 2020 pre-pandemic

© 2024 Yang and Toriola. *JAMA Health Forum.*

eTable 10.1 Weighted logistic regression of menopausal hormone therapy use among US postmenopausal women by race/ethnicity, 1999-2018[a]

	Odds Ratio (95% CI)[b]				
	**Overall**	**Non-Hispanic White**	**Non-Hispanic Black**	**Hispanic**	**Others**
**Age**	0.96 (0.95 to 0.97)	0.96 (0.95 to 0.97)	0.98 (0.96 to 0.99)	0.98 (0.96 to 1.00)	0.94 (0.89 to 1.00)
**Race/ethnicity**					
Non-Hispanic white	1 [Reference]				
Non-Hispanic black	0.45 (0.35 to 0.57)				
Hispanic	0.46 (0.35 to 0.60)				
Other[c]	0.53 (0.34 to 0.83)				
**Family income to poverty ratio**					
<1.3	1 [Reference]	1 [Reference]	1 [Reference]	1 [Reference]	1 [Reference]
1.3 - <3.5	1.59 (1.24 to 2.03)	1.58 (1.17 to 2.13)	1.55 (0.84 to 2.86)	1.16 (0.66 to 2.04)	3.47 (0.87 to 13.75)
>=3.5	2.15 (1.65 to 2.80)	2.08 (1.52 to 2.85)	1.90 (1.13 to 3.21)	2.11 (1.14 to 3.93)	6.60 (1.18 to 36.90)
Missing	1.12 (0.77 to 1.64)	1.11 (0.71 to 1.74)	1.48 (0.53 to 4.16)	0.86 (0.39 to 1.91)	1.28 (0.12 to 14.27)
**Educational attainment**					
<High school	1 [Reference]	1 [Reference]	1 [Reference]	1 [Reference]	1 [Reference]
High school	1.01 (0.79 to 1.28)	0.87 (0.65 to 1.18)	2.36 (1.27 to 4.39)	0.69 (0.37 to 1.29)	2.60 (0.58 to 11.67)
>High school	1.19 (0.95 to 1.48)	1.01 (0.77 to 1.31)	3.34 (2.02 to 5.51)	1.81 (1.10 to 2.97)	2.07 (0.48 to 8.83)
**Health insurance**					
No insurance	1 [Reference]	1 [Reference]	1 [Reference]	1 [Reference]	1 [Reference]
Any insurance	2.06 (1.37 to 3.11)	1.75 (1.10 to 2.80)	3.71 (1.54 to 8.95)	3.06 (1.11 to 8.43)	1.60 (0.52 to 4.96)
Private insurance	1.96 (1.36 to 2.83)	1.65 (1.09 to 2.50)	3.47 (1.41 to 8.50)	3.91 (1.69 to 9.01)	NA
Missing	0.97 (0.27 to 3.50)	0.86 (0.10 to 7.09)	2.21 (0.19 to 25.49)	1.86 (0.33 to 10.51)	NA
**Marital status**					
Living alone	1 [Reference]	1 [Reference]	1 [Reference]	1 [Reference]	1 [Reference]
Living with someone	1.15 (0.96 to 1.39)	1.15 (0.94 to 1.41)	1.61 (1.08 to 2.40)	1.19 (0.78 to 1.82)	0.54 (0.20 to 1.50)
Missing	0.48 (0.27 to 0.83)	0.35 (0.17 to 0.70)	0.52 (0.07 to 3.68)	2.14 (0.29 to 5.55)	5.22 (0.33 to 2.27)
**Body mass index**					
<25 kg/m^2	1 [Reference]	1 [Reference]	1 [Reference]	1 [Reference]	1 [Reference]
25-<30 kg/m^2	0.99 (0.79 to 1.23)	0.93 (0.72 to 1.19)	1.15 (0.67 to 1.97)	1.51 (0.83 to 2.77)	2.28 (0.76 to 6.85)
≥30 kg/m^2	0.69 (0.55 to 0.85)	0.64 (0.50 to 0.82)	0.84 (0.49 to 1.41)	1.01 (0.53 to 1.91)	1.67 (0.50 to 5.58)
Missing	0.65 (0.47 to 0.91)	0.62 (0.43 to 0.88)	0.44 (0.16 to 1.25)	1.28 (0.43 to 3.82)	1.25 (0.21 to 7.56)
**Smoke status**					
Never	1 [Reference]	1 [Reference]	1 [Reference]	1 [Reference]	1 [Reference]
Past	1.16 (0.96 to 1.39)	1.12 (0.91 to 1.37)	1.28 (0.81 to 2.03)	1.17 (0.73 to 1.88)	1.64 (0.62 to 4.29)
Current	0.85 (0.67 to 1.08)	0.76 (0.58 to 0.98)	1.17 (0.70 to 1.96)	2.03 (1.09 to 3.79)	0.68 (0.22 to 2.06)
Missing	1.45 (0.16 to 2.85)	1.43 (0.12 to 6.81)		7.68 (1.12 to 2.45)	NA
**Study cycle**					

© 2024 Yang and Toriola. *JAMA Health Forum.*

1999-2000	1 [Reference]		1 [Reference]		1 [Reference]		1 [Reference]		1 [Reference]	
2001-2002	0.95	(0.68 to 1.33)	0.91	(0.64 to 1.27)	1.27	(0.59 to 2.74)	0.97	(0.41 to 2.28)	2.00	(0.21 to 18.73)
2003-2004	0.43	(0.33 to 0.57)	0.40	(0.30 to 0.54)	0.75	(0.38 to 1.48)	0.57	(0.19 to 1.75)	0.90	(0.10 to 8.14)
2005-2006	0.29	(0.21 to 0.39)	0.28	(0.20 to 0.38)	0.39	(0.14 to 1.07)	0.26	(0.07 to 1.06)	0.45	(0.04 to 5.65)
2007-2008	0.23	(0.17 to 0.33)	0.22	(0.15 to 0.32)	0.17	(0.06 to 0.43)	0.37	(0.15 to 0.90)	0.64	(0.07 to 6.12)
2009-2010	0.15	(0.10 to 0.23)	0.15	(0.10 to 0.23)	0.14	(0.06 to 0.36)	0.14	(0.05 to 0.40)	0.23	(0.02 to 2.69)
2011-2012	0.18	(0.13 to 0.27)	0.19	(0.13 to 0.27)	0.15	(0.07 to 0.32)	0.34	(0.11 to 1.03)	0.05	(0.00 to 0.71)
2013-2014	0.21	(0.14 to 0.31)	0.20	(0.13 to 0.31)	0.29	(0.11 to 0.75)	0.24	(0.09 to 0.63)	0.48	(0.06 to 4.21)
2015-2016	0.16	(0.09 to 0.27)	0.15	(0.09 to 0.26)	0.08	(0.02 to 0.28)	0.13	(0.04 to 0.42)	0.76	(0.06 to 9.16)
2017-2018	0.15	(0.09 to 0.23)	0.15	(0.09 to 0.24)	0.06	(0.02 to 0.17)	0.15	(0.05 to 0.43)	0.16	(0.02 to 1.50)
***P* for trend**[d]	<0.001		<0.001		<0.001		<0.001		0.017	

a All data are weighted to be US nationally representative.

b Odds ratios (ORs) represent the change in odds expected in each category compared with the reference group.

c “Other” race/ethnicity includes race/ethnicity other than non-Hispanic white, non-Hispanic Black and Hispanic, including multiracial.

d *P* for trend over study cycle was calculated using the National Health and National Examination Survey (NHANES) study 2-year survey cycle as a continuous variable.

© 2024 Yang and Toriola. *JAMA Health Forum.*

eTable 10.2 Weighted logistic regression of menopausal hormone therapy use among US postmenopausal women with no history of breast cancer, 1999-March 2020 pre-pandemic[a]

	Odds ratio (95% CI)[b]				
	**Overall**	**Non-Hispanic White**	**Non-Hispanic Black**	**Hispanic**	**Others**
**Age**	0.96 (0.95 to 0.97)	0.96 (0.95 to 0.97)	0.98 (0.96 to 1.00)	0.98 (0.96 to 1.00)	0.95 (0.89 to 1.01)
**Race/ethnicity**					
Non-Hispanic white	1 [Reference]				
Non-Hispanic black	0.44 (0.34 to 0.56)				
Hispanic	0.45 (0.34 to 0.59)				
Other[c]	0.53 (0.34 to 0.82)				
**Family income to poverty ratio**					
<1.3	1 [Reference]	1 [Reference]	1 [Reference]	1 [Reference]	1 [Reference]
1.3 - <3.5	1.60 (1.24 to 2.05)	1.59 (1.17 to 2.15)	1.53 (0.82 to 2.85)	1.18 (0.66 to 2.11)	3.43 (0.85 to 13.90)
≥3.5	2.19 (1.67 to 2.86)	2.11 (1.53 to 2.91)	1.87 (1.11 to 3.16)	2.19 (1.17 to 4.11)	7.24 (1.27 to 41.17)
Missing	1.13 (0.77 to 1.67)	1.13 (0.71 to 1.78)	1.42 (0.50 to 4.05)	0.88 (0.40 to 1.96)	1.26 (0.11 to 14.31)
**Educational attainment**					
<High school	1 [Reference]	1 [Reference]	1 [Reference]	1 [Reference]	1 [Reference]
High school	1.00 (0.78 to 1.27)	0.86 (0.64 to 1.17)	2.55 (1.38 to 4.71)	0.70 (0.38 to 1.31)	2.37 (0.53 to 10.54)
>High school	1.17 (0.94 to 1.46)	0.98 (0.75 to 1.29)	3.52 (2.17 to 5.72)	1.82 (1.10 to 3.01)	2.08 (0.51 to 8.50)
**Health insurance**					
No insurance	1 [Reference]	1 [Reference]	1 [Reference]	1 [Reference]	1 [Reference]
Any insurance	2.08 (1.38 to 3.14)	1.78 (1.11 to 2.83)	3.50 (1.45 to 8.47)	3.14 (1.17 to 8.43)	1.62 (0.53 to 5.00)
Private insurance	1.98 (1.37 to 2.86)	1.67 (1.10 to 2.54)	3.43 (1.40 to 8.39)	3.84 (1.68 to 8.80)	NA
Missing	0.97 (0.27 to 3.50)	0.82 (0.10 to 6.72)	2.24 (0.20 to 24.99)	2.15 (0.35 to 13.20)	NA
**Marital status**					
Living alone	1 [Reference]	1 [Reference]	1 [Reference]	1 [Reference]	1 [Reference]
Living with someone	1.13 (0.94 to 1.35)	1.13 (0.93 to 1.37)	1.57 (1.06 to 2.32)	1.11 (0.72 to 1.71)	0.46 (0.17 to 1.25)
Missing	0.46 (0.26 to 0.80)	0.34 (0.17 to 0.68)	0.47 (0.06 to 3.34)	2.29 (0.32 to 16.58)	5.16 (0.34 to 79.46)
**Body mass index**					
<25 kg/m^2	1 [Reference]	1 [Reference]	1 [Reference]	1 [Reference]	1 [Reference]
25-<30 kg/m^2	0.97 (0.79 to 1.20)	0.91 (0.71 to 1.15)	1.18 (0.69 to 2.03)	1.55 (0.85 to 2.83)	2.21 (0.72 to 6.78)
≥30 kg/m^2	0.66 (0.54 to 0.81)	0.61 (0.48 to 0.78)	0.85 (0.50 to 1.43)	0.88 (0.46 to 1.67)	1.82 (0.57 to 5.75)
Missing	0.61 (0.44 to 0.85)	0.58 (0.41 to 0.83)	0.44 (0.15 to 1.28)	1.19 (0.41 to 3.49)	0.97 (0.17 to 5.56)
**Smoke status**					
Never	1 [Reference]	1 [Reference]	1 [Reference]	1 [Reference]	1 [Reference]
Past	1.16 (0.97 to 1.40)	1.12 (0.91 to 1.37)	1.29 (0.81 to 2.04)	1.24 (0.80 to 1.92)	1.70 (0.68 to 4.27)
Current	0.85 (0.67 to 1.09)	0.75 (0.58 to 0.99)	1.08 (0.64 to 1.84)	1.97 (1.06 to 3.68)	0.69 (0.22 to 2.15)
Missing	1.36 (0.15 to 11.95)	1.35 (0.12 to 15.80)	NA	7.88 (1.15 to 54.08)	NA
**Study cycle**					

© 2024 Yang and Toriola. *JAMA Health Forum.*

1999-2000	1 [Reference]			1 [Reference]			1 [Reference]			1 [Reference]			1 [Reference]		
2001-2002	0.95	(0.68 to	1.32)	0.91	(0.65 to	1.27)	1.16	(0.54 to	2.51)	0.96	(0.41 to	2.24)	2.12	(0.22 to	20.23)
2003-2004	0.43	(0.33 to	0.55)	0.39	(0.30 to	0.52)	0.74	(0.36 to	1.49)	0.57	(0.18 to	1.76)	0.97	(0.11 to	8.90)
2005-2006	0.29	(0.22 to	0.39)	0.28	(0.20 to	0.38)	0.38	(0.14 to	1.05)	0.26	(0.07 to	1.06)	0.43	(0.03 to	5.59)
2007-2008	0.24	(0.17 to	0.33)	0.22	(0.16 to	0.32)	0.17	(0.06 to	0.43)	0.39	(0.16 to	0.96)	0.63	(0.07 to	6.02)
2009-2010	0.15	(0.10 to	0.23)	0.15	(0.10 to	0.23)	0.14	(0.05 to	0.35)	0.14	(0.05 to	0.40)	0.24	(0.02 to	2.75)
2011-2012	0.18	(0.12 to	0.26)	0.18	(0.12 to	0.26)	0.14	(0.06 to	0.32)	0.36	(0.11 to	1.10)	0.05	(0.00 to	0.72)
2013-2014	0.21	(0.15 to	0.31)	0.20	(0.13 to	0.30)	0.29	(0.11 to	0.76)	0.25	(0.10 to	0.65)	0.51	(0.06 to	4.56)
2015-2016	0.16	(0.09 to	0.28)	0.16	(0.09 to	0.26)	0.08	(0.02 to	0.28)	0.13	(0.04 to	0.44)	0.75	(0.06 to	9.03)
2017-2020	0.11	(0.07 to	0.17)	0.11	(0.07 to	0.18)	0.03	(0.01 to	0.07)	0.14	(0.05 to	0.35)	0.16	(0.02 to	1.35)
***P* for trend**[d]	<0.001			<0.001			0.003			0.002			0.002		

a All data are weighted to be US nationally representative.

b Odds ratios (ORs) represent the change in odds expected in each category compared with the reference group.

c "Other" race/ethnicity includes race/ethnicity other than non-Hispanic white, non-Hispanic Black and Hispanic, including multiracial.

d *P* for trend over study cycle was calculated using the National Health and National Examination Survey (NHANES) study 2-year survey cycle as a continuous variable.

© 2024 Yang and Toriola. *JAMA Health Forum.*

eTable 10.3 Weighted logistic regression of menopausal hormone therapy use among US postmenopausal women with complete data on correlates, 1999-March 2020 pre-pandemic[a]

	Odds ratio (95% CI)[b]				
	**Overall**	**Non-Hispanic White**	**Non-Hispanic Black**	**Hispanic**	**Others**
**Age**	0.96 (0.95 to 0.97)	0.96 (0.95 to 0.97)	0.97 (0.95 to 0.99)	0.98 (0.95 to 1.00)	0.94 (0.88 to 1.01)
**Race/ethnicity**					
Non-Hispanic white	1 [Reference]				
Non-Hispanic black	0.45 (0.34 to 0.59)				
Hispanic	0.45 (0.33 to 0.61)				
Other[c]	0.53 (0.32 to 0.87)				
**Family income to poverty ratio**					
<1.3	1 [Reference]	1 [Reference]	1 [Reference]	1 [Reference]	1 [Reference]
1.3 - <3.5	1.56 (1.19 to 2.06)	1.54 (1.11 to 2.13)	1.71 (0.87 to 3.37)	1.22 (0.67 to 2.24)	3.47 (0.76 to 15.79)
≥3.5	2.17 (1.63 to 2.88)	2.06 (1.48 to 2.88)	2.17 (1.19 to 3.96)	2.32 (1.17 to 4.57)	7.28 (1.11 to 47.54)
P for trend					
**Educational attainment**					
<High school	1 [Reference]	1 [Reference]	1 [Reference]	1 [Reference]	1 [Reference]
High school	1.09 (0.84 to 1.41)	0.99 (0.71 to 1.37)	2.59 (1.32 to 5.09)	0.67 (0.35 to 1.30)	1.49 (0.35 to 6.43)
>High school	1.23 (0.97 to 1.57)	1.08 (0.81 to 1.45)	3.47 (1.97 to 6.12)	1.46 (0.85 to 2.50)	1.42 (0.36 to 5.53)
P for trend					
**Health insurance**					
No insurance	1 [Reference]	1 [Reference]	1 [Reference]	1 [Reference]	1 [Reference]
Any insurance	2.20 (1.40 to 3.43)	1.86 (1.12 to 3.11)	4.62 (1.73 to 12.34)	2.89 (1.09 to 7.66)	1.83 (0.57 to 5.86)
Private insurance	2.06 (1.38 to 3.09)	1.78 (1.12 to 2.84)	4.13 (1.58 to 10.79)	3.38 (1.43 to 7.95)	NA
**Marital status**					
Living alone	1 [Reference]	1 [Reference]	1 [Reference]	1 [Reference]	1 [Reference]
Living with someone	1.10 (0.90 to 1.34)	1.09 (0.89 to 1.34)	1.43 (0.90 to 2.28)	1.12 (0.69 to 1.82)	0.55 (0.18 to 1.68)
**Body mass index**					
<25 kg/m^2	1 [Reference]	1 [Reference]	1 [Reference]	1 [Reference]	1 [Reference]
25-<30 kg/m^2	1.07 (0.86 to 1.34)	1.00 (0.78 to 1.28)	1.30 (0.72 to 2.35)	1.83 (1.01 to 3.33)	1.99 (0.58 to 6.83)
≥30 kg/m^2	0.73 (0.60 to 0.90)	0.68 (0.54 to 0.86)	0.98 (0.58 to 1.67)	0.92 (0.48 to 1.78)	1.96 (0.56 to 6.86)
P for trend					
**Smoke status**					
Never	1 [Reference]	1 [Reference]	1 [Reference]	1 [Reference]	1 [Reference]
Past	1.16 (0.95 to 1.41)	1.13 (0.91 to 1.40)	1.19 (0.75 to 1.90)	0.92 (0.54 to 1.58)	2.65 (1.01 to 6.94)
Current	0.78 (0.60 to 1.01)	0.68 (0.51 to 0.91)	1.31 (0.75 to 2.28)	2.11 (1.16 to 3.85)	0.59 (0.16 to 2.19)
**Study cycle**					
1999-2000	1 [Reference]	1 [Reference]	1 [Reference]	1 [Reference]	1 [Reference]
2001-2002	0.92 (0.64 to 1.32)	0.83 (0.57 to 1.22)	1.71 (0.72 to 4.06)	1.19 (0.51 to 2.78)	2.95 (0.31 to 27.89)

© 2024 Yang and Toriola. *JAMA Health Forum.*

2003-2004	0.44	(0.34 to 0.58)	0.40	(0.30 to 0.53)	0.90	(0.37 to 2.20)	0.70	(0.22 to 2.17)	1.09	(0.12 to 9.51)
2005-2006	0.30	(0.22 to 0.41)	0.28	(0.20 to 0.39)	0.52	(0.18 to 1.51)	0.32	(0.08 to 1.31)	0.41	(0.03 to 6.40)
2007-2008	0.23	(0.16 to 0.33)	0.22	(0.15 to 0.32)	0.19	(0.06 to 0.63)	0.46	(0.19 to 1.13)	0.55	(0.05 to 5.55)
2009-2010	0.15	(0.11 to 0.23)	0.15	(0.10 to 0.24)	0.20	(0.08 to 0.53)	0.13	(0.04 to 0.40)	0.05	(0.00 to 0.70)
2011-2012	0.19	(0.13 to 0.27)	0.18	(0.13 to 0.26)	0.21	(0.09 to 0.51)	0.44	(0.14 to 1.37)	0.05	(0.00 to 0.71)
2013-2014	0.21	(0.14 to 0.31)	0.19	(0.13 to 0.30)	0.39	(0.14 to 1.10)	0.31	(0.12 to 0.79)	0.41	(0.05 to 3.58)
2015-2016	0.16	(0.09 to 0.28)	0.15	(0.08 to 0.25)	0.11	(0.03 to 0.44)	0.11	(0.02 to 0.55)	0.86	(0.08 to 9.06)
2017-2020	0.12	(0.08 to 0.19)	0.13	(0.08 to 0.20)	0.03	(0.01 to 0.12)	0.17	(0.07 to 0.42)	0.11	(0.01 to 0.90)
***P* for trend**[d]		<0.001		<0.001		0.026		0.004		0.009

a All data are weighted to be US nationally representative.

b Odds ratios (ORs) represent the change in odds expected in each category compared with the reference group.

c “Other” race/ethnicity includes race/ethnicity other than non-Hispanic white, non-Hispanic Black and Hispanic, including multiracial.

d *P* for trend over study cycle was calculated using the National Health and National Examination Survey (NHANES) study 2-year survey cycle as a continuous variable.

© 2024 Yang and Toriola. *JAMA Health Forum.*
