# Supplementary material for: Menopausal Hormone Therapy Use Among Postmenopausal Women
Source: JAMA Health Forum. 2024 Sep 27;5(9):e243128. doi: 10.1001/jamahealthforum.2024.3128 (PMC11437377; doi:10.1001/jamahealthforum.2024.3128)
Supplement: Supplement 2. — Data Sharing Statement [file jamahealthforum-e243128-s002.pdf]

Data Sharing Statement

Yang. Menopausal Hormone Therapy Use Among Postmenopausal Women. *JAMA Health Forum*. Published September 27, 2024. doi:10.1001/jamahealthforum.2024.3128

Data
Data available: Yes
Data types: Deidentified participant data
How to access data: Data are publicly available via https://www.cdc.gov/nchs/nhanes/index.htm
When available: With publication

Supporting Documents
Document types: Other (please specify)
Additional Information: Data and statistical/analytic guides are publicly available via https://www.cdc.gov/nchs/nhanes/index.htm
How to access documents: Data and statistical/analytic guides are publicly available via https://www.cdc.gov/nchs/nhanes/index.htm
When available: With publication

Additional Information
Who can access the data: Data are publicly available via https://www.cdc.gov/nchs/nhanes/index.htm
Types of analyses: Data are publicly available via https://www.cdc.gov/nchs/nhanes/index.htm
Mechanisms of data availability: Data are publicly available via https://www.cdc.gov/nchs/nhanes/index.htm
